# Design, Synthesis and Biological Evaluation of C(6)-Modified Celastrol Derivatives as Potential Antitumor Agents

**DOI:** 10.3390/molecules190710177

**Published:** 2014-07-14

**Authors:** Kaiyong Tang, Qingqing Huang, Jafeng Zeng, Guangming Wu, Jinwen Huang, Junfang Pan, Wei Lu

**Affiliations:** 1 Institute of Drug Discovery and Development, Shanghai Engineering Research Center of Molecular Therapeutics and New Drug Development, East China Normal University, Shanghai 200062, China; E-Mails: tangkaiyong@huatuodrug.com (K.T.); qqhuang@sat.ecnu.edu.cn (Q.H.); 2 Shanghai Hotmed Sciences Co., Ltd., Shanghai 201201, China; E-Mails: zengjiafeng@huatuodrug.com (J.Z.); wuguangming@huatuodrug.com (G.W.); huangjinwen@hotmail.com (J.H.)

**Keywords:** celastrol C-6 derivatives, antitumor activity, *in vitro*, *in vivo*

## Abstract

New six C6-celastrol derivatives were designed, synthesized, and evaluated for their *in vitro* cytotoxic activities against nine human cancer cell lines (BGC-823, H4, Bel7402, H522, Colo 205, HepG2 and MDA-MB-468). The results showed that most of the compounds displayed potent inhibition against BGC823, H4, and Bel7402, with IC_50_s of 1.84–0.39 μM. The best compound NST001A was tested in an *in vivo* antitumor assay on nude mice bearing Colo 205 xenografts, and showed significant inhibition of tumor growth at low concentrations. Therefore, celastrol C-6 derivatives are potential drug candidates for treating cancer.

## 1. Introduction

**Figure 1:**
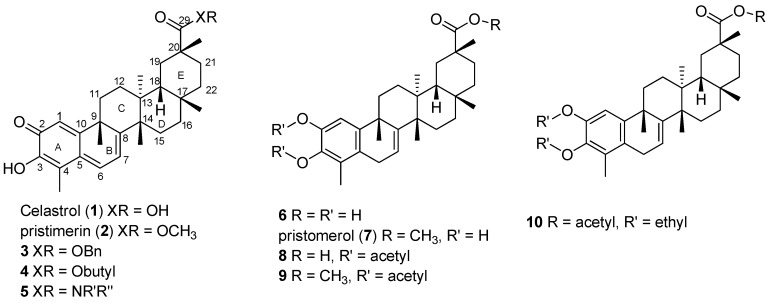
Structures of celastrol and its analogues.

Celastrol (tripterine, **1**, Figure 1), a quinone methide triterpene, was extracted from the Traditional Chinese Medicine “Thunder of God Vine” (*Tripterygium wilfordii* Hook F.). Recently, abundant research has indicated that celastrol is involved with several novel molecular targets, such as Hsp90-Cdc37, NF-κB/IKKβ, VEGFR1, and VEGFR2 [1,2], which suggest its development as an antitumor, antifungal, antioxidant and anti-inflammatory agent. Celastrol is also known to inhibit the proliferation of various tumor cells, including those of gliomas [3], hepatocellular carcinomas [4] and prostate cancer [5]. In addition, it potently inhibited the proteasome and induced apoptosis [5,6,7], suggesting it might serve as a potential cancer treatment.

Although celastrol is very effective toward many molecular targets, it has some deficiencies, such as poor stability, poor water solubility and high toxicity, which hinder its further application and thus has attracted intensive interest from medicinal scientists. Some structure modifications were reported in order to improve solubility or reduce toxicity, a simple strategy being the esterification or amidification of 20-carboxylic acid of celastrol, such as seen in compounds **2**–**5** (Figure 1), that lead to decreased potency [8,9,10]. Some analogues with modified A/B rings at the C-2, 3 positions (**6**–**10**, Figure 1) were reported as the inducers of heat shock response [8,9,10]. However, the mechanism of action underlying the anti-inflammatory and antiproliferative effects of celastrol and most analogues is not yet fully understood.

**Scheme 1:**
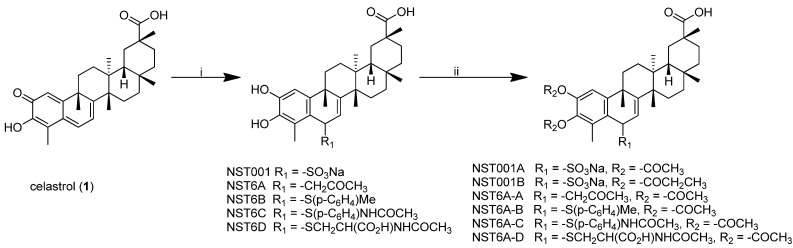
Synthetic scheme for the designed compounds.

Our company, Shanghai Hotmed Sciences Co. Ltd., is one of the biggest suppliers of celastrol in China, In a previous study [11], we patented some water soluble analogues with improved potency modified at the C-6 position of celastrol. **NST001**, a C(6)-sulfonated analogues derived from the reaction of celastrol and bisulfate sodium (Scheme 1), was selected for animal test evaluation, in which it displayed good activity on PC-3 tumor-bearing nude mice (i.p. 3 mg/kg, dl/3.5 w) and the toxicity was decreased by compared with celastrol [11]. These promising results suggested the C-6 position of celastrol as a potentially useful modification site. In order to explore more the chemical space and obtain potential drug candidates with improved pharmacologic profiles and low toxicity, six novel C(6)-modified celastrol analogues were prepared and are reported herein.

## 2. Results and Discussion

### 2.1. Synthesis of Celastrol C-6 Analogues (Scheme 1)

**NST001** was a white solid recrystallized from ethanol/water binary solution. Acetylation of the two hydroxyl groups of **NST001** affords **NST001A**. **NST6A-A** was synthesized from the reaction of celastrol and acetone catalyzed by 1 N HCl, followed by acetylation. **NST6A** (**B**–**D**) were obtained from the reaction of celastrol and mercaptans (or thiophenols), followed by acetylation. All compounds were identified by ^1^H-NMR, ^13^C-NMR, IR, LC-ESI-MS, [α], and the HPLC purity of all compounds was over 98.0%.

*Reagents and conditions:* (i) For **NST001**: NaHSO_3_, MeOH, RT, 1.5 h, N_2_; For **NST6A**: acetone, 1 N HCl (cat.), RT, N_2_; For **NST6A~D**: RSH, MeOH, RT, N_2_; (ii) Ac_2_O, Py, RT, N_2_; or (CH_3_CH_2_CO)_2_O, Py, RT, N_2_.

### 2.2. Activity of Celastrol Analogues against Human Cancer Cell Lines

**Figure 2:**
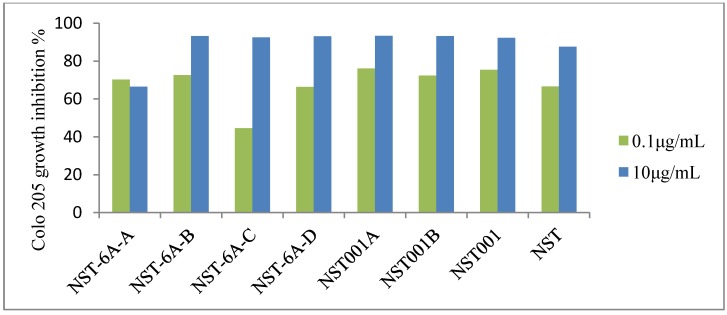
The inhibitory rate (at concentrations of 0.1, 10 μg/mL for 72 h) on Colo 205 growth. The results are expressed as the percentage of that of the treat cells.

To evaluated the antiproliferative effects of celastrol analogues on human cancer cells, all compounds were screened against a variety of human cancer cell lines including the human gastric cancer cell line BGC-823, and the human liver cancer cell lines H4 and Bel7402. As shown in Table 1, compounds bearing a C–S bond showed similar or better inhibition than celastrol (**1**), however, the compound bearing a C–C bond at the C-6 position (**NST6A-A**) completely lost its activity. To investigate the inhibition on more sensitive human colon cancer cell line Colo 205, another cytotoxicity assay was conducted at concentrations of 0.1, 10 μg/mL, respectively. As shown in Figure 2, the compound **NST6A-A** displayed decreased cytotoxicity at a concentration of 10 μg/mL. The cytotoxicity of other analogues with C(6)-sulfided celastrol showed good dose-response relationships. Among them, sodium bisulfate modifications (**NST001**, **NST001A**, **NST001B**) showed prospective effects against Colo 205 growth.

**Table 1:** IC_50_ values (μM) on human cancer cell lines.

Compounds	IC_50_ (μM)		
	BGC823	H4	Bel7402
**Celastrol (1)**	3.73	2.09	1.55
**NST001**	0.77	0.91	1.17
**NST001A**	0.49	1.37	1.73
**NST001B**	0.74	1.12	2.39
**NST6A-A**	>150	>150	>150
**NST6A-B**	0.47	0.37	0.45
**NST6A-C**	0.42	0.35	0.46
**NST6A-D**	0.50	0.46	0.54

As **NST001** and **NST001A** showed the most potent inhibition at both concentrations, these two compounds were selected to identify precise IC_50_ values on the human non-small cell lung cell line H522, human colon cancer cell line Colo 205, human hepatocellular liver carcinoma cell line HepG2, human breast adenocarcinoma cell line MDA-MB-468 and human gastric cancer cell line BGC823, respectively. The results are summarized in Table 2. **NST001A** was more potent than NST001 in anti-tumor profile except for the BGC823 cell line. Interestingly, Colo 205 cells are more sensitive to **NST001A** than the other four cancer cell types, and that compound shows good selectivity to Colo 205 cells with potency increased about 8- times compared to the parent compound **NST001**. 

**Table 2:** IC_50_ values (μM) of **NST001** and **NST001A** on human cancer cell lines.

Compounds	IC_50_ (μM) for 72 h				
	H522	Colo 205	HepG2	MDA-MB-468	BGC823
**NST001A**	0.39	0.06	0.29	0.33	1.53
**NST001**	0.47	0.51	0.68	0.89	0.77

### 2.3. In Vivo Anti-Tumor Activity Evaluation

**Figure 3:**
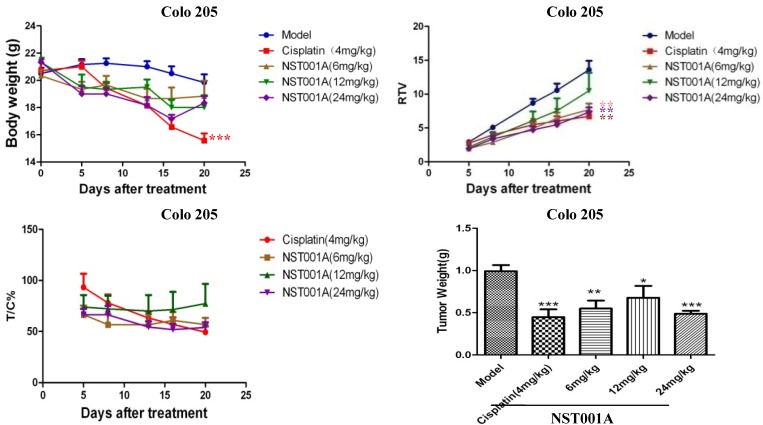
Effects of **NST001A** on the growth of Colo 205 xenografts in nude mice.

As shown above, **NST001A** was more potent than the other C6-derivatives and the most active compound on Colo 205 cells. Thus, female nude mice loaded Colo 205 xenografts were used for *in vivo* anti-tumor activity evaluation of the most potent compound **NST001A**. The body weights of the nude mice were monitored and their tumor sizes were measured and recorded. The rate of inhibition of the different groups against Colo 205 solid tumors in female nude mice are shown in Figure 3. After treatment, there were no apparent body weight loss during the treatment in the negative control group injected with saline solution (Model), and the negative control group induced an average tumor weight of 0.99 ± 0.25 g, the T/C (%) value treated with cisplatin (4 mg/kg, i.p., qd × 20) was 49.32% with about 6 g body weight lost. The **NST001A**-treated group showed similar activity as cisplatin, the T/C (%) value of **NST001A** ranged from 54.00% to 77.25% without obvious body weight loss. These results proved that **NST001A** was a potential antitumor drug candidate with lower toxicity compared with cisplatin.

All date are means ± SD: * *p* < 0.05, ** *p* < 0.01, *** *p* < 0.001 when compared with model.

Tumor size and body weight were measured and clinical symptoms were observed at day 0, 5, 8, 13, 16, 20. The tumor volume (TV) was calculated according to the formula: TV = (1/2 × length × width^2^), and the relative tumor volume (RTV) was calculated according to the formula: RTV = TV_t_/TV_0_, where TV_t_ is the tumor volume at day t, and TV_0_ is the tumor volume at the first treatment. The relative growth rate of the tumor T/C (%) calculated as follows:

[0066] T/C (%) = RTVt/RTVc × 100%T/C (%) = (T_RTV_/C_RTV_) × 100%

where the T_RTV_ is the treatment group relative tumor volume; C_RTV_ is the control group relative tumor volume. Data are expressed as means ± SD. Statistical evaluations were analyzed with one-way ANOVA (*t*-test). *p* < 0.05 was considered statistically significant.

### 2.4. Preliminary Non-Clinical Safety Evaluation

**Figure 4:**
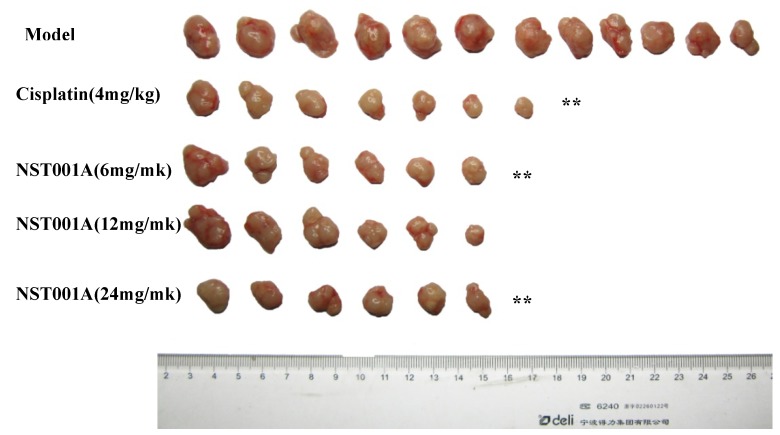
Tumor growth after systemic application of different drug loaded systems.

The *in vivo* anti-cancer evaluation showed that **NST001A** was a potential antitumor compound, and safer than cisplatin (4 mg/kg). Thus, **NST001A** along with **NST001** were evaluated for preliminary non-clinical safety evaluation on female nude mice through injection in the tail vein for seven days (i.v., qd × 7). The results are shown in Figure 4. After loading for 7 days, the body weight of the control group ranged from 17.0 g up to 19.0 g, and all the test groups lost a certain weight. During the experiment, the injection site of **NST001** group showed successive swelling and fracture, but this phenomenon was not found in the **NST001A** groups. Polypnea was found in one mouse in the test with **NST001A** (40 mg/kg) and disappeared after drug withdrawal. After delivery for 7 days, all mice were put to death, and no obvious tissue lesions were found in any of the groups. These results suggested that **NST001A** was safer than **NST001**.

All date are means ± SD: ** *p* < 0.01, when compared with model.

## 3. Experimental Section

### 3.1. General Information

Celastrol was extracted from the Traditional Chinese Medicine (*Tripterygium wilfordii* Hook F.) by ourselves. Silica gel FCP200-300 mesh was used for column chromatography. All chemicals were purchased from Sinopharm Chemical Reagent Co., Ltd. (Shanghai, China). ^1^H-NMR and ^13^C-NMR spectra were collected in CDCl_3_ and DMSO-*d*_6_ at 25 °C on a Bruker AV-400 spectrometer. All chemical shifts are reported in the standard δ notation of parts per million using the peak of residual proton signals of CDCl_3_ or DMSO-*d*_6_ as an internal reference. Electrospray ionization (ESI) analyses were performed by using a Thermo LTQ (LC-ESI-MS/MS). Melting point analyses were performed by using a digital micro-melting point apparatus. All compounds were confirmed by ^1^H-NMR, ^13^C-NMR, IR, LC-ESI-MS, [α], and the HPLC purity of all compounds was over 98.0%. All the *in vitro* and *in vivo* tests were carried out in accordance with guidelines evaluated and approved by the Shanghai Institute of Materia Medica Chinese Academy of Sciences (SIMM). All animal experiments were performed under specific pathogen-free conditions in accordance with institutional guidelines.

### 3.2. Synthesis

*Sodium (6bS,8aS,11R,12aR,12bS,14aS)-2,3-diacetoxy-11-carboxy-4,6b,8a,11,12b,14a-hexamethyl-5,6b,7,8,8a,9,10,11,12,12a,12b,13,14,14a-tetradecahydropicene-5-sulfonate* (**NST001**). To a stirred solution of celastrol (100 mg, 0.22 mmol) in methanol (5 mL) was added a solution of sodium bisulfate (27 mg, 0.26 mmol in 2 mL water) under nitrogen. After 1.5 h, the reaction solution concentrated and dried under vacuum at 40 °C, recrystallization from ethanol/water afforded the white compound **NST001** (103 mg, 84% yield). HPLC purity > 98.0% (mobile phase 78:22 (v/v) mixture of methanol and 0.005% H_3_PO_4_). ^1^H-NMR (DMSO-*d*_6_): δ 12.01 (s, 1H), 8.81 (s, 1H), 7.62 (s, 1H), 6.58 (s, 3H), 5.81 (d, *J* = 6.2 Hz, 1H), 4.49 (d, *J* = 6.2 Hz, 1H), 2.21 (s, 3H), 1.62 (s, 3H), 1.18 (s, 3H), 1.09 (s, 3H), 1.05 (s, 3H), 0.59 (s, 3H).

*Sodium (6bS,8aS,11R,12aR,12bS,14aS)-2,3-diacetoxy-11-carboxy-4,6b,8a,11,12b,14a-hexamethyl-5,6b,7,8,8a,9,10,11,12,12a,12b,13,14,14a-tetradecahydropicene-5-sulfonate* (**NST001A**). To a solution of **NST001** (100 mg, 0.18 mmol) in pyridine (3 mL) was added acetic anhydride (0.1 mL) at room temperature under nitrogen. After stirring for 3 h, the mixture was quenched with ethanol (5 mL). The solvent was removed under reduced pressure, the residue was purified by gel filtration chromatography (anhydrous ethanol) to afford the title compound as a white solid (80 mg, 69% yield). HPLC purity > 98.0% (mobile phase 60:40 (v/v) mixture of acetonitrile and 0.005% H_3_PO_4_). Mp > 160 °C. 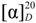 −94.76° (MeOH, c = 0.2). ^1^H-NMR (DMSO-*d*_6_): δ 7.01 (s, 1H), 5.83 (d, *J* = 6.1 Hz, 1H), 4.60 (d, *J* = 6.0 Hz, 1H), 2.50 (s, 1H), 2.29 (s, 2H), 2.24 (d, *J* = 6.7 Hz, 5H), 2.08 (s, 1H), 1.99 (dd, *J* = 23.8, 12.5 Hz, 3H), 1.83 (dd, *J* = 21.7, 15.3 Hz, 1H), 1.67 (s, 2H), 1.63–1.36 (m, 8H), 1.28 (s, 1H), 1.20 (s, 3H), 1.09 (s, 3H), 1.05 (s, 2H), 0.85 (d, *J* = 13.8 Hz, 1H), 0.61 (s, 2H). ^13^C-NMR (DMSO-*d*_6_): δ 179.9, 168.9, 168.7, 149.0, 148.6, 141.3, 138.5, 130.5, 129.8, 118.5, 117.3, 60.1, 56.5, 44.3, 44.1, 38.8, 37.8, 36.9, 36.6, 35.1, 34.4, 32.9, 31.9, 30.6, 30.5, 29.9, 29.1, 21.6, 20.9, 20.6, 19.0, 18.6, 13.8. IR (KBr) 3447.9, 2944.5, 2873.1, 1768.8, 1466.0, 1374.3, 1228.7, 1194.0, 1158.3, 1039.7 cm^−1^. LC-ESI-MS (−) *m/z* (%) = 615.41 [M−1Na^+^]^−^ (100%), 1231.86 [2M−2Na^+^]^2−^ (10%).

*Sodium (6bS,8aS,11R,12aR,12bS,14aS)-11-carboxy-4,6b,8a,11,12b,14a-hexamethyl-2,3-bis-(propionyloxy)-5,6b,7,8,8a,9,10,11,12,12a,12b,13,14,14a-tetradecahydropicene-5-sulfonate* (**NST001B**). To a solution of **NST001** (100 mg, 0.18 mmol) in pyridine (3 mL) was added propionic anhydride (0.12 mL) at room temperature under nitrogen. After stirred for 3 h, the mixture was quenched with ethanol (5 mL). The solvent was removed under reduced pressure, the residue was purified by gel filtration chromatography (anhydrous ethanol) to afford the title compound as a white solid (86 mg, 71.6% yield). HPLC purity > 98.0% (mobile phase 55:45 (v/v) mixture of acetonitrile and 0.005% H_3_PO_4_). mp > 150 °C. 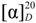 −98.21° (MeOH, c = 0.2). ^1^H-NMR (DMSO-d_6_): δ 7.01 (s, 1H), 5.83 (d, *J* = 6.2 Hz, 1H), 4.60 (d, *J* = 6.1 Hz, 1H), 2.66–2.45 (m, 3H), 2.36 (d, *J* = 14.7 Hz, 1H), 2.24 (s, 1H), 2.10 (d, *J* = 12.8 Hz, 1H), 2.06–1.93 (m, 1H), 1.82 (d, *J* = 5.7 Hz, 1H), 1.68 (s, 2H), 1.40 (d, *J* = 15.0 Hz, 4H), 1.22 (d, *J* = 14.1 Hz, 2H), 1.17–1.07 (m, 4H), 1.05 (s, 3H), 0.83 (d, *J* = 11.8 Hz, 1H), 0.64 (s, 2H). ^13^C-NMR (DMSO-*d*_6_): δ 180.6, 172.1, 171.9, 149.0, 148.7, 141.3, 138.3, 130.4, 129.7, 118.3, 117.2, 60.0, 56.3, 44.3, 44.0, 38.7, 37.8, 37.0, 36.7, 35.2, 34.5, 33.2, 32.6, 31.9, 30.6, 30.4, 30.2, 30.0, 29.1, 27.1, 26.9, 21.6, 18.5, 13.8, 9.6, 9.4. IR (KBr): 2969.5, 2944.6, 2873.1, 1762.0, 1559.5, 1457.3, 1155.4, 1040.6 cm^−1^. LC-ESI-MS (−) *m/z* (%) = 643.45 [M−1Na^+^]^−^ (100%), 1288.09 [2M−2Na^+^]^2−^ (5%).

*(2R,4aS,6aS,12bS,14aS,14bR)-10,11-diacetoxy-2,4a,6a,9,12b,14a-hexamethyl-8-(2-oxopropyl)-1,2,3,4,4a,5,6,6a,8,12b,13,14,14a,14b-tetradecahydropicene-2-carboxylic acid* (**NST6A-A**). To a solution of celastrol (130 mg, 0.29 mmol) in acetone (10 mL) was added 1 drop of 1 N HCl. The mixture was stirred overnight under nitrogen, then the solution concentrated under vacuum. The residue was redissolved in pyridine (0.4 mL), and acetic anhydride (2 mL) were added. The mixture was stirred overnight under nitrogen. The reaction was quenched with 3 mL ice water, then the mixture was extracted with ethyl acetate, concentrated under vacuum. The residue was purified by silica gel (petroleum ether/acetone) as a pale solid (123 mg, 72% yield). HPLC purity > 98.0% (mobile phase 87:13 (v/v) mixture of acetonitrile and 0.005% H_3_PO_4_); mp 146.3–147.5 °C; 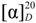 −92.16° (MeOH, c = 0.2); ^1^H-NMR (CDCl_3_): δ 7.00 (s, 1H), 5.85 (d, *J* = 6.0 Hz, 1H), 4.25–3.86 (m, 1H), 2.36 (t, *J* = 11.1 Hz, 1H), 2.32 (s, 3H), 2.29 (s, 3H), 2.15 (s, 3H), 2.09 (m, 4H), 1.88–1.57 (m, 5H), 1.53 (d, *J* = 4.3 Hz, 1H), 1.50 (s, 3H), 1.42 (dd, *J* = 16.3, 5.1 Hz, 3H), 1.27 (s, 3H), 1.20 (s, 3H), 1.10 (s, 3H), 1.06 (s, 3H), 0.93–0.83 (m, 4H), 0.66 (s, 3H); ^13^C-NMR (CDCl_3_): δ 207.2, 184.1, 168.5, 168.2, 149.7, 148.2, 140.8, 138.7, 134.1, 127.4, 121.3, 117.3, 53.7, 51.4, 44.2, 43.8, 40.1, 37.6, 36.6, 35.4, 34.5, 33.0, 32.6, 31.5, 30.8, 30.4, 30.1, 29.4, 28.8, 22.6, 22.4, 20.7, 20.4, 18.6, 12.1; IR (KBr) 2941.6, 2872.1, 1768.8, 1465.0, 1369.5, 1213.3, 1188.2 cm^−1^; LC-ESI-MS (−) *m/z* (%) = 591.3 [M−1H^+^]^−^ (5%), 1183.3 [2M−1H^+^]^−^ (100%), 1775.8 [3M−1H^+^]^−^ (60%). 

*(2R,4aS,6aS,12bS,14aS,14bR)-10,11-diacetoxy-2,4a,6a,9,12b,14a-hexamethyl-8-(p-tolylthio)-1,2,3,4,4a,5,6,6a,8,12b,13,14,14a,14b-tetradecahydropicene-2-carboxylic acid* (**NST6A-B**). To a solution of celastrol (100 mg, 0.22 mmol) in methanol (3 mL), *p*-thiocresol (40 mg, 0.32 mmol) was added, the reation was stirred for 1 h at room temperature under nitrogen, then the reaction mixture was concentrated under reduced pressure, and the residue was redissolved in pyridine (0.5 mL) and acetic anhydride (4 mL) was added, the reaction was stirred overnight under nitrogen. Then quenched by ice water, extracted with ethyl acetate, concentrated under vacuum and purified by chromatography on silica gel (petroleum ether/acetone) as a white solid (97 mg, 67% yield). HPLC purity > 98.0% (mobile phase 80:20 (v/v) mixture of acetonitrile and 0.005% H_3_PO_4_). mp 109.4–110.3 °C. 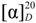 −69.82° (MeOH, c = 0.2). ^1^H-NMR (CDCl_3_) δ 7.34 (d, *J* = 7.9 Hz, 1H), 7.11 (d, *J* = 7.8 Hz, 1H), 7.01 (s, 1H), 5.70 (d, *J* = 6.1 Hz, 1H), 4.75 (d, *J* = 6.0 Hz, 1H), 2.34 (s, 2H), 2.32 (s, 1H), 2.30 (s, 1H), 2.28 (s, 1H), 1.47 (s, 1H), 1.32 (d, *J* = 11.9 Hz, 3H), 1.25 (s, 3H), 1.22 (s, 1H), 1.09 (s, 2H), 1.04 (s, 1H), 0.96 (t, *J* = 7.4 Hz, 1H), 0.87 (d, *J* = 7.1 Hz, 1H), 0.60 (s, 3H); ^13^C-NMR (DMSO-*d*_6_): δ 179.8, 168.9, 168.7, 152.0, 149.7, 142.1, 139.1, 138.0, 133.7, 130.9, 130.2, 130.1, 129.6, 119.9, 117.8, 44.8, 44.1, 44.1, 38.2, 37.6, 36.7, 35.0, 34.8, 34.1, 32.7, 31.8, 30.6, 30.4, 29.9, 29.8, 29.5, 28.8, 22.3, 21.2, 20.9, 20.5, 18.6, 12.3; IR (KBr) 2924.2, 2870.2, 1772.7, 1696.5, 1490.1, 1369.5, 1210.4, 1188.2 cm^−1^. LC-ESI-MS (−) *m/z* (%) = 657.1 [M−1H^+^]^−^ (6%), 1315.2 [2M−1H^+^]^−^ (100%), 1927.8 [3M−1H^+^]^−^ (65%).

*(2R,4aS,6aS,12bS,14aS,14bR)-10,11-diacetoxy-8-((4-aminophenyl)thio)-2,4a,6a,9,12b,14a-hexamethyl-1,2,3,4,4a,5,6,6a,8,12b,13,14,14a,14b-tetradecahydropicene-2-carboxylic acid* (**NST6A-C**). A solution of celastrol (100 mg, 0.22 mmol) and 4-acetamidothiophenol (50 mg, 0.30 mmol) in methanol (3 mL) was stirred for 1hat room temperature under nitrogen, the solvent was removed under reduced pressure. The residue was redissolved in acetic anhydride (4 mL) and pyridine (0.5 mL) was added and stirred overnight under nitrogen. The reaction solution was quenched by ice water, extracted with ethyl acetate, concentrated under vacuum and purified by chromatography on silica gel (petroleum ether/acetone) as a white solid (84 mg, 54% yield). HPLC purity > 98.0% (mobile phase 80:20 (v/v) mixture of acetonitrile and 0.005% H_3_PO_4_); mp 157–160 °C; 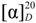 −66.23° (MeOH, c = 0.2). ^1^H-NMR (CDCl_3_) δ 7.76 (d, *J* = 22.2 Hz, 1H), 7.37–7.21 (m, 4H), 7.01 (s, 1H), 5.59 (d, *J* = 6.1 Hz, 1H), 4.51 (d, *J* = 6.0 Hz, 1H), 3.47 (d, *J* = 2.9 Hz, 1H), 2.39 (s, 1H), 2.34 (d, *J* = 7.0 Hz, 7H), 2.30 (s, 3H), 2.12 (t, *J* = 7.4 Hz, 4H), 2.02 (d, *J* = 13.1 Hz, 2H), 1.88–1.54 (m, 6H), 1.50 (s, 1H), 1.47 (s, 3H), 1.43–1.29 (m, 4H), 1.25 (s, 2H), 1.21 (s, 3H), 1.14 (s, 3H), 1.05 (s, 3H), 0.94–0.82 (m, 1H), 0.53 (s, 3H); ^13^C-NMR (DMSO-*d*_6_): δ 180.6, 179.8, 168.9, 168.7, 151.9, 149.7, 142.0, 139.9, 139.1, 135.0, 130.1, 129.6, 127.4, 119.9, 119.7, 117.8, 60.0, 45.2, 44.1, 44.1, 38.2, 37.7, 36.7, 35.0, 34.8, 34.1, 32.7, 31.8, 30.6, 30.4, 30.0, 29.8, 28.8, 24.5, 22.3, 20.9, 20.5, 18.6, 12.4; IR (KBr) 2940.6, 2871.2, 1775.5, 1700.2, 1590.3, 1524.8, 1369.5, 1360.8, 1213.3 cm^−1^. LC-ESI-MS (−) *m/z* (%) = 700.9 [M−1H^+^]^−^ (7%), 746.0 [M+2Na^+^−1H^+^]^+^ (55%), 1402.1 [2M−1H^+^]^−^ (15%), 1447.5 [2M+2Na^+^−1H^+^]^+^ (100%).

*(2R,4aS,6aS,12bS,14aS,14bR)-8-((2-acetamido-2-carboxyethyl)thio)-10,11-diacetoxy-2,4a,6a,9,12b,14a-hexamethyl-1,2,3,4,4a,5,6,6a,8,12b,13,14,14a,14b-tetradecahydropicene-2-carboxylic acid* (**NST6A-D**). A solution of celastrol (200 mg, 0.44 mmol) and acetylcysteine (91 mg, 0.56 mmol) methanol (5 mL), was stirred at room temperature under nitrogen. After 1 h, the solvent was removed under reduced pressure, and the residue was redissolved in acetic anhydride (0.15 mL) and pyridine (3 mL) was added, the reaction was stirred for 1 h under nitrogen, then quenched by ice water, extracted with ethyl acetate, concentrated under vacuum and purified by chromatography on silica gel (ethyl acetate) as a pale solid (81 mg, 26% yield). HPLC purity > 98.0% ( mobile phase 70:30 (v/v) mixture of acetonitrile and 0.005% H_3_PO_4_); mp 187–189 °C; 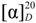 −111.01° (MeOH, c = 0.2); ^1^H-NMR (DMSO-*d*_6_) δ 8.14–7.78 (m, 1H), 7.09 (s, 1H), 6.09 (dd, *J* = 24.2, 5.7 Hz, 1H), 4.71 (d, *J* = 5.4 Hz, 1H), 4.38 (s, 1H), 4.27 (s, 1H), 3.56–3.53 (m, 1H), 3.44 (dd, *J* = 13.8, 6.9 Hz, 1H), 3.24 (d, *J* = 9.5 Hz, 1H), 3.06 (d, *J* = 5.5 Hz, 1H), 2.76 (dd, *J* = 13.3, 8.2 Hz, 1H), 2.50 (s, 1H), 2.33 (d, *J* = 16.1 Hz, 2H), 2.24 (s, 1H), 2.19 (d, *J* = 9.8 Hz, 1H), 2.16–1.92 (m, 2H), 1.86 (s, 1H), 1.83 (s, 1H), 1.63 (d, *J* = 9.2 Hz, 2H), 1.55–1.36 (m, 3H), 1.25 (d, *J* = 24.4 Hz, 3H), 1.10 (s, 2H), 1.05 (d, *J* = 9.1 Hz, 2H), 0.87 (d, *J* = 11.2 Hz, 1H), 0.64 (s, 2H); ^13^C-NMR (DMSO-*d*_6_): δ 179.9, 172.8, 169.2, 169.0, 168.9, 168.7, 152.0, 151.6, 149.5, 149.4, 141.8, 141.7, 138.9, 131.4, 131.2, 129.4, 129.4, 120.3, 119.8, 117.5, 117.4, 56.4, 54.6, 53.8, 45.7, 44.3, 44.2, 38.1, 38.1, 37.6, 36.8, 35.0, 34.8, 34.2, 34.2, 34.0, 33.9, 32.8, 31.8, 31.7, 30.6, 30.4, 29.9, 29.9, 28.6, 23.2, 23.2, 22.6, 22.5, 22.4, 22.3, 21.7, 20.9, 20.5, 19.0, 18.8, 12.3, 12.1; IR (KBr) 2939.6, 2871.2, 1771.69, 1374.3, 1213.3 cm^−1^; LC-ESI-MS (−) *m/z* (%) = 696.4 [M−1H^+^]^−^ (8%), 1393.5 [2M−1H^+^]^−^ (100%).

### 3.3. Activity of Celastrol Analogues against Human Cancer Cell Lines

*In vitro* culture, BGC-823, H4, Bel7402, H522, Colo 205, HepG2, MDA-MB-468 cells grown to logarithmic growth phase were collected, and centrifuged at 1,000 rpm for 5 min. The cells were seeded at a density of 3.5 × 10^4^/mL in 96-well plate (100 μL/well), incubator (37 °C, 5% CO_2_) for 24 h, the test drugs at serial dilutions were added, the negative control group was added to a final concentration of 0.5% DMSO, After the incubator for another 72 h, added to each well of 5 mg/mL MTT (20 μL/well) and cultured for 4 h, then DMSO was added in (150 μL/well), shaking for 5 min and the absorbance were measured at 492 nm and 620 nm. The growth inhibition rate was calculated by the software GraphPad Prism.

### 3.4. Anti-Tumor Activity Evaluation and Preliminary Non-Clinical Safety Evaluation in Vivo

BALB/c nude mice (16–17 g), female, 4–6 weeks, supplied by Shanghai SLAC Laboratory Animal Co. Ltd. (Shanghai, China, License No:SCXK (Hu)2007-0005). Cisplatin for injection were supplied by Qilu-pharma (Jinan, China), Normal saline were supplied by Jiangsu Shihuan Bioengineering Co., Ltd (Wuxi, China), human colon cancer cell Colo 205 was purchased from ATCC.

For the *in vivo* anti-tumor activity evaluation, the exuberant growth of human colon cancer Colo 205 tumor tissue was cut into 1.5 mm^3^ tumor fragments, and inoculated into nude mice subcutaneously under aseptic conditions. When tumors reached an average volume of 100–150 mm^3^, the nude mice were randomly divided into five groups according to the experimental plan (Table 3).

**Table 3:** The experimental plan of tumor bearing nude mice treated with NST001A.

Groups	N.O. of Mice	Dosage		Route of Administration	Defined Daily Dose
		(mg/kg)	(mL/10 g)		
Model	12	0.2 mL	0.1	i.p.	qd × 20
Cisplatin (4 mg/kg)	6	4		i.p.	3 times/week
NST001A (6 mg/kg)	6	6		i.p.	qd × 20
NST001A (12 mg/kg)	6	12		i.p.	qd × 20
NST001A (24 mg/kg)	6	24		i.p.	qd × 20

**Figure 5:**
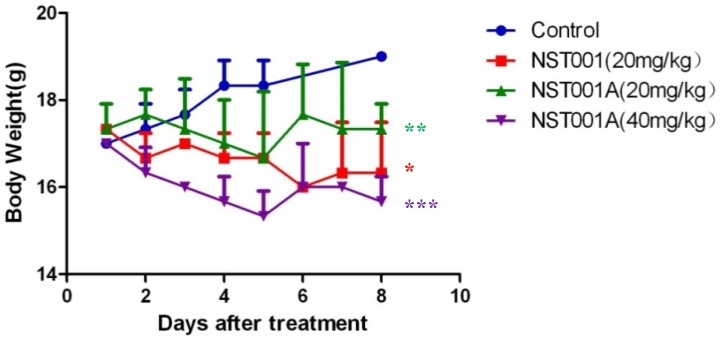
Effects of different drug loaded system on the body weight of nude mice.

Female nude mice were used for preliminary non-clinical safety evaluation of **NST001A**. The compound was soluble in normal saline for injecting in the tail vein for seven days (i.v., qd × 7). The results are shown in Table 4 and Figure 5.

**Table 4:** The change of nude mice bodyweight of different drug loaded systems.

Group	N.O. of Mice		Body Weight (g)	
	Initial	Final	Initial	Final
control	3	3	17.0 ± 0.00	19.00 ± 0.00
NST001 (20 mg/kg)	3	3	17.33 ± 0.58	16.33 ± 1.15 *
NST001A (20 mg/kg)	3	3	17.33 ± 0.58	17.33 ± 0.58 **
NST001A (40 mg/kg)	3	3	17.0 ± 0.00	15.67 ± 0.58 ***

* *p* < 0.05, ** *p* < 0.01, *** *p* < 0.001 when compared with model.

All data are means ± SD: * *p* < 0.05, ** *p* < 0.01, *** *p* < 0.001 when compared with control.

## 4. Conclusions

In summary, we have synthesized three kinds of C(6)-modified celastrol derivatives and their anticancer activity were assayed at the cell level. Comparing with the parent compound celastrol, the *in vitro* cytotoxicity potency of C(6)-sulfonted and C(6)-sulfided celastrol derivatives increased about 5–8 times against the human cancer cell BGC823, H4, Bel7401, however, for the C(6)–carbonated celastol derivatives, the potency dropped dramatically. Moreover, 2,3-acetylation was more effective than propionylation. The best compound, **NST001A**, which shows good selectivity towards Colo 205 cell with a 0.06 μM IC_50_ value, was selected for *in vivo* evaluation in nude mice bearing xenografts of human colon cancer. The results indicated that **NST001A** can potently inhibit the growth of Colo 205 xenografts in nude mice with a better safety profile compared with **NST001**.

## Supplementary Materials

Supplementary materials can be accessed at:  http://www.mdpi.com/1420-3049/19/7/10177/s1.
