# Supplementary material for: Design, Synthesis and Biological Evaluation of C(6)-Modified Celastrol Derivatives as Potential Antitumor Agents
Source: Molecules. 2014 Jul 14;19(7):10177–88. doi: 10.3390/molecules190710177 (PMC6271447; doi:10.3390/molecules190710177)
Supplement: Supplementary file 1 [file molecules-19-10177-s001.pdf]

Supplementary Information

Contents

Figure S1. IC_{50} values (μg/mL) of celastrol and 1 analogues on BGC823. .... S2
Figure S2. IC_{50} values (μg/mL) of celastrol and 1 analogues on H4. .... S3
Figure S3. IC_{50} values (μg/mL) of celastrol and 1 analogues on Bel7402. .... S4
Figure S4. IC_{50} values (μg/mL) of NST001A on human cancer cell lines. S5
Figure S5. IC_{50} values (μg/mL) of NST001 on human cancer cell lines. S5
Table S1. The Mw of the Compounds (g/mol) S6
Figure S6. The inhibitory rate (at concentrations of 0.1, 10 μg/mL for 72 h) on Colo 205 growth. The results are expressed as the percentage of that of the treat cells. S6
Scheme S1. Synthetic scheme of the designed compounds. S7
Table S2. The change of the mice body weight and the inhibitory rate of tumor(T/C) of different drug loaded system against Colo 205 tumor in nude mice (mean ± SD). S8
Figure S7. Effects of NST001A on the growth of Colo 205 xenografts in nude mice. S8
^{1}H-NMR (NST001A) S9
^{1}H-NMR (NST001B) S9
^{1}H-NMR (NST6A-A) S10
^{1}H-NMR (NST6A-B) S10
^{1}H-NMR (NST6A-C) S11
^{1}H-NMR (NST6A-D) S11
^{13}C-NMR (NST001A) S12
^{13}C-NMR (NST00B) S12
^{13}C-NMR (NST6A-A) S13
^{13}C-NMR (NST6A-B) S13
^{13}C-NMR (NST6A-C) S14
^{13}C-NMR (NST6A-D) S14
LC-ESI-MS(−) (NST001A) S15
LC-ESI-MS(−) (NST001B) S15
LC-ESI-MS(−) (NST6A-A) S16
LC-ESI-MS(−) (NST6A-B) S16
LC-ESI-MS(−) (NST6A-C) S17
LC-ESI-MS(−) (NST6A-D) S17
HPLC purity (NST001) S18
HPLC purity (NST001A) S19
HPLC purity (NST001B) S20
HPLC purity (NST6A-A) S21
HPLC purity (NST6A-B) S22
HPLC purity (NST6A-C) S23
HPLC purity (NST6A-D) S24

Figure S1. IC_{50} values (μg/mL) of celastrol and 1 analogues on BGC823.

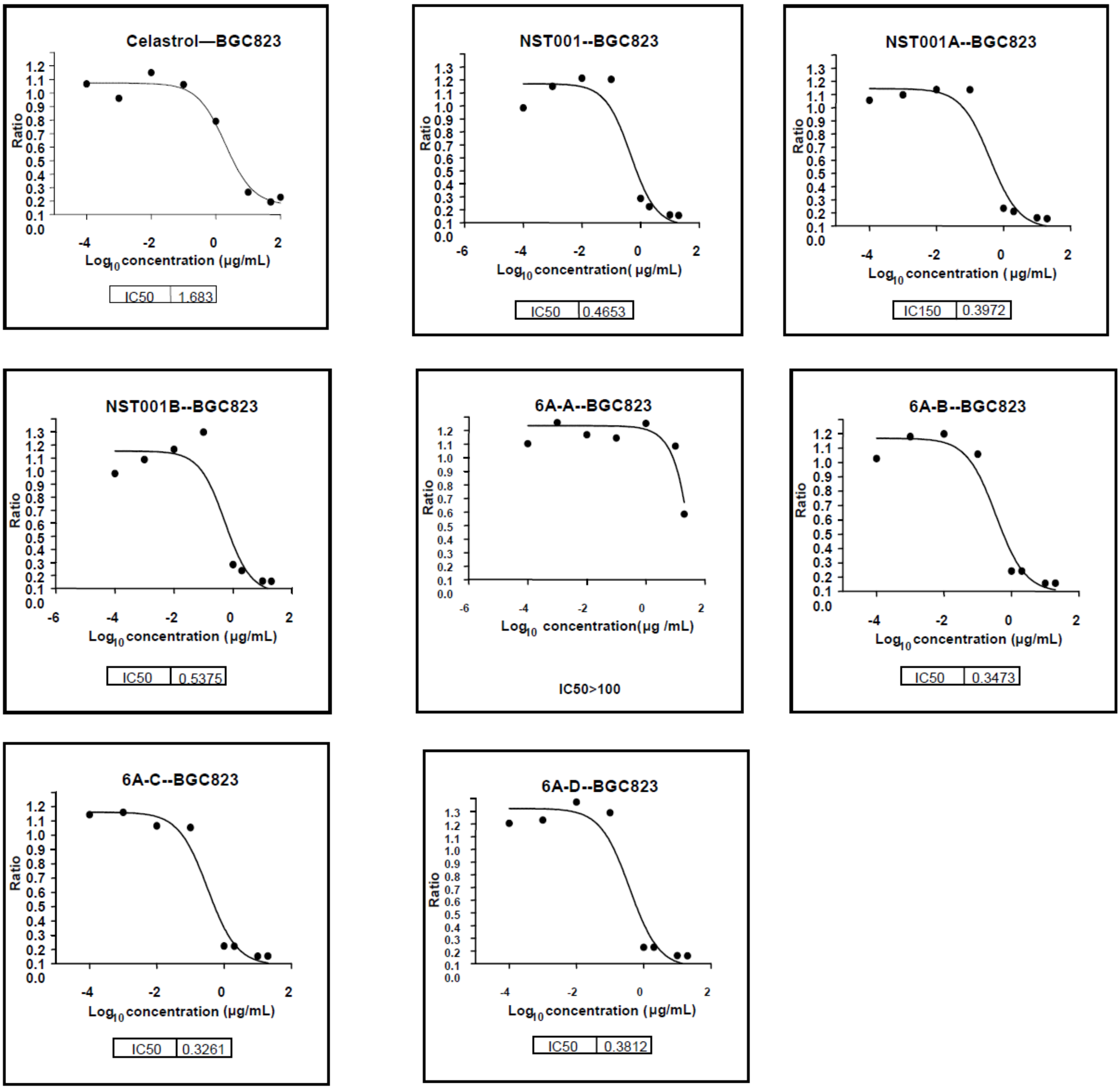

Figure S2. IC_{50} values (μg/mL) of celastrol and 1 analogues on H4.

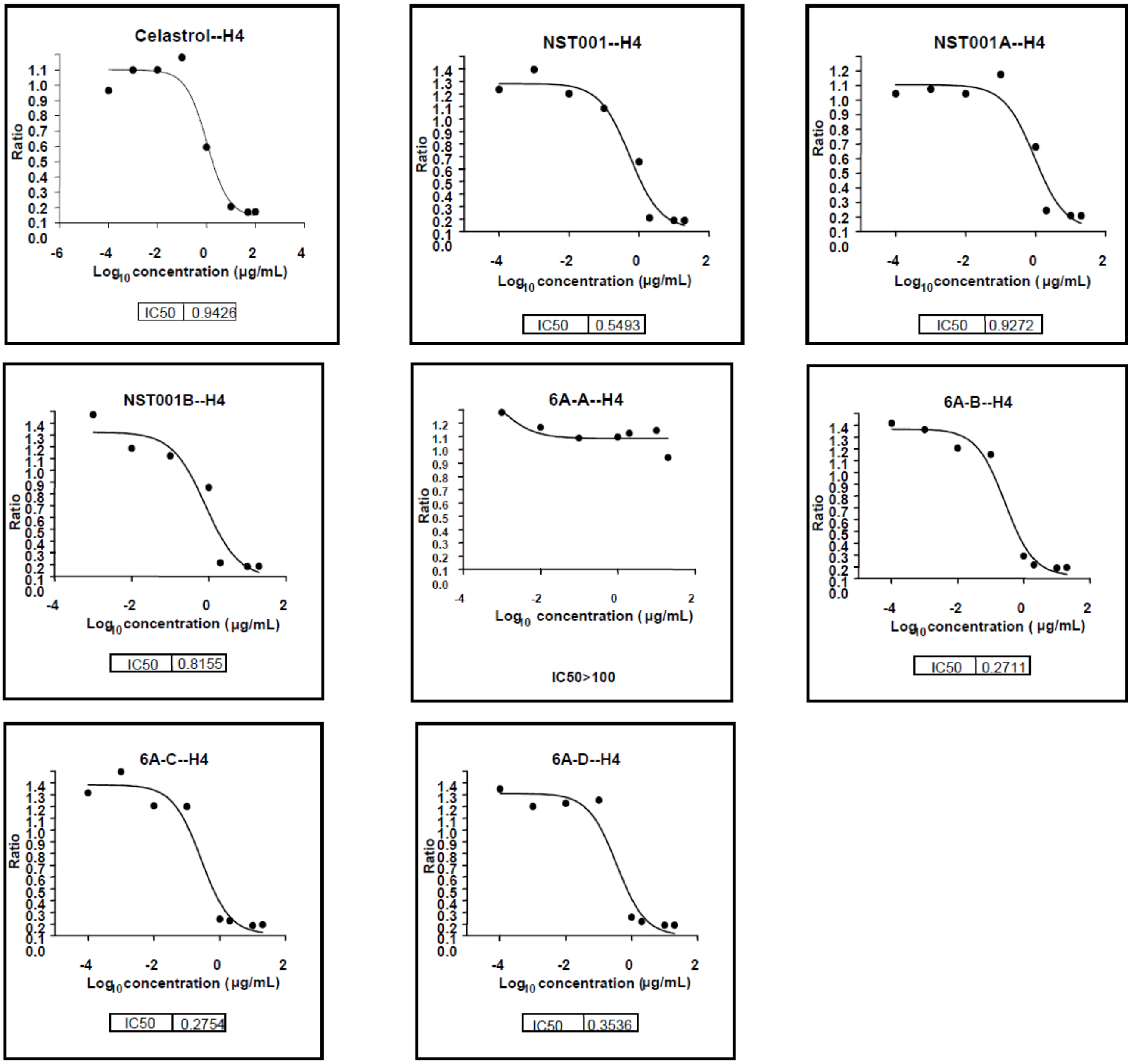

Figure S3. IC_{50} values (μg/mL) of celastrol and 1 analogues on Bel7402.

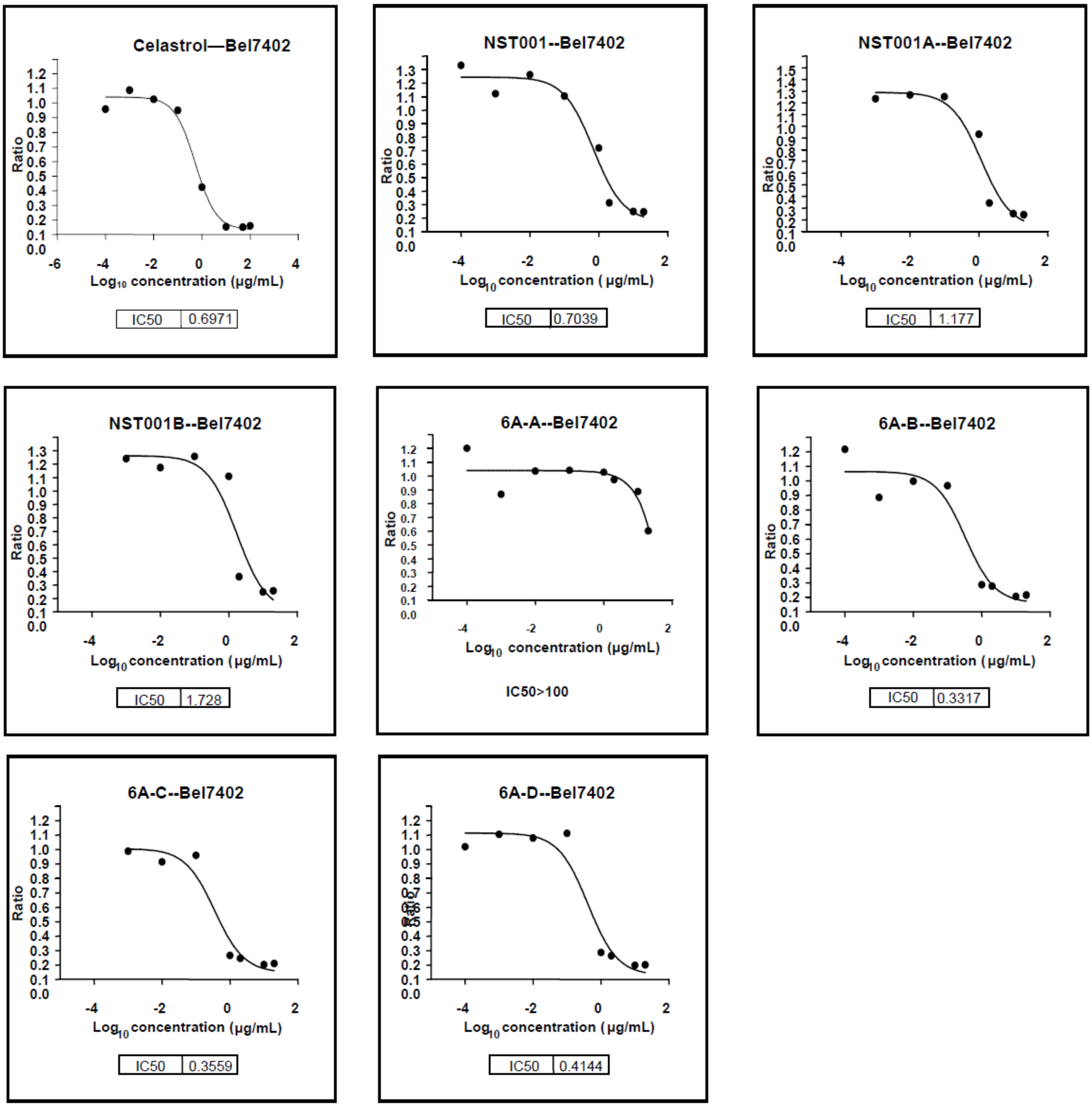

Figure S4. IC_{50} values (μg/mL) of NST001A on human cancer cell lines.

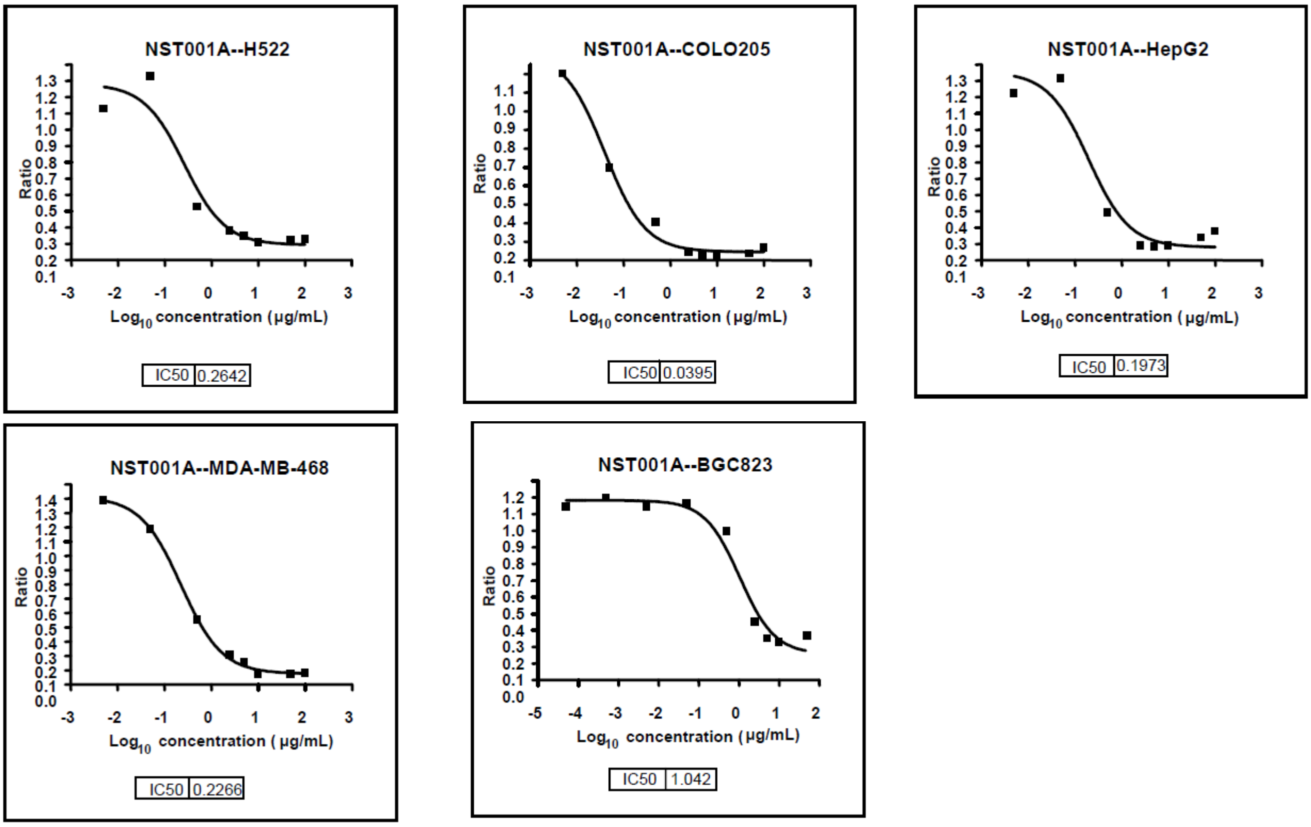

Figure S5. IC_{50} values (μg/mL) of NST001 on human cancer cell lines.

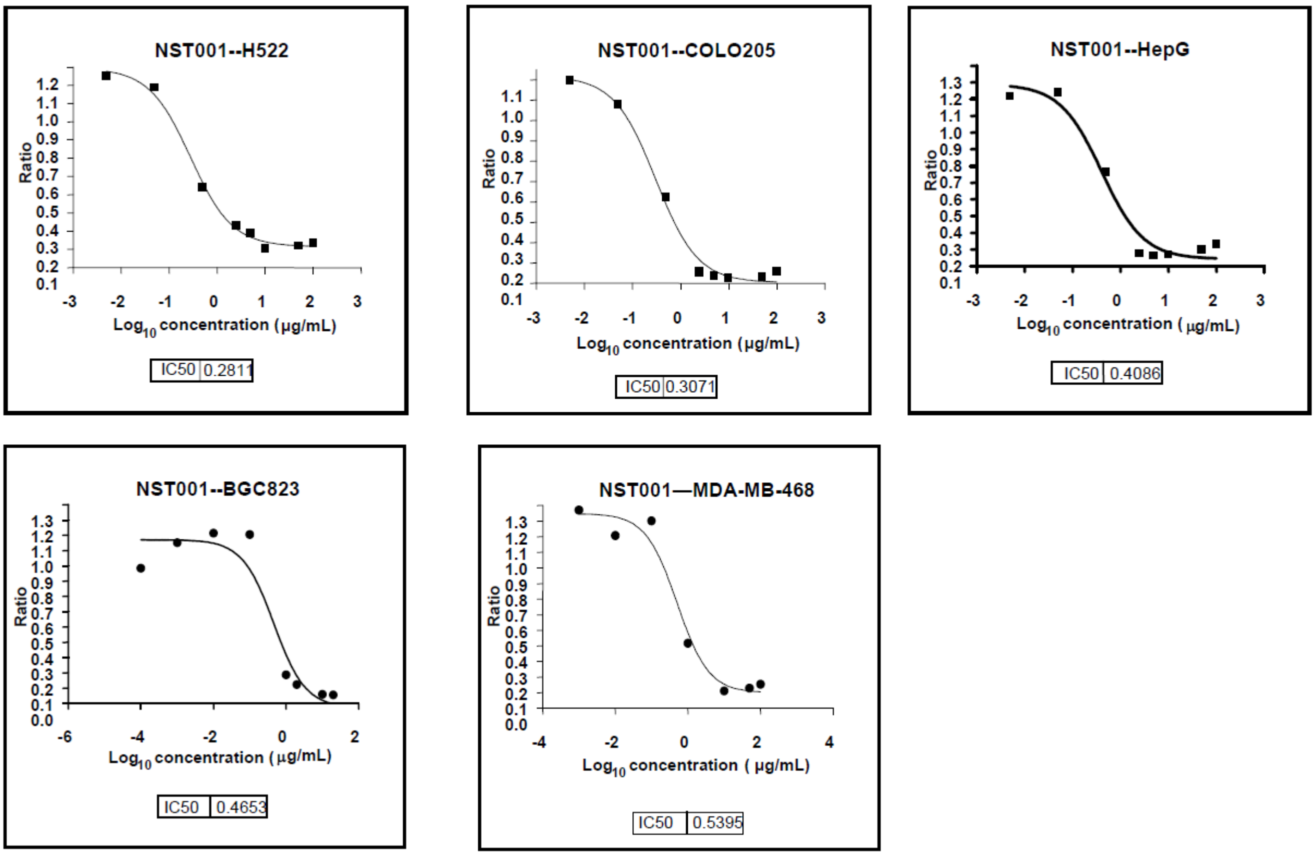

The IC_{50} values (μM) in the article was calculated according to the following formula: IC_{50} (μM) = IC_{50} (μg/mL)/Mw × 1000, the molecular weight (Mw) was list in the table.

Table S1. The Mw of the Compounds (g/mol).

NST	NST001	NST001B	NST001A	NST-6A-B	NST-6A-C	NST-6A-D
450.61	603.67	726.7	678.83	733.87	778.09	764.06

Figure S6. The inhibitory rate (at concentrations of 0.1, 10 μg/mL for 72 h) on Colo 205 growth. The results are expressed as the percentage of that of the treat cells.

	NST-6A-A	NST-6A-B	NST-6A-C	NST-6A-D	NST001A	NST001B	NST001	NST
10 μg/mL	66.51%	93.24%	92.50%	93.16%	93.32%	93.27%	92.34%	87.54%
0.1 μg/mL	70.26%	72.52%	44.55%	66.35%	76.12%	72.29%	75.45%	66.65%

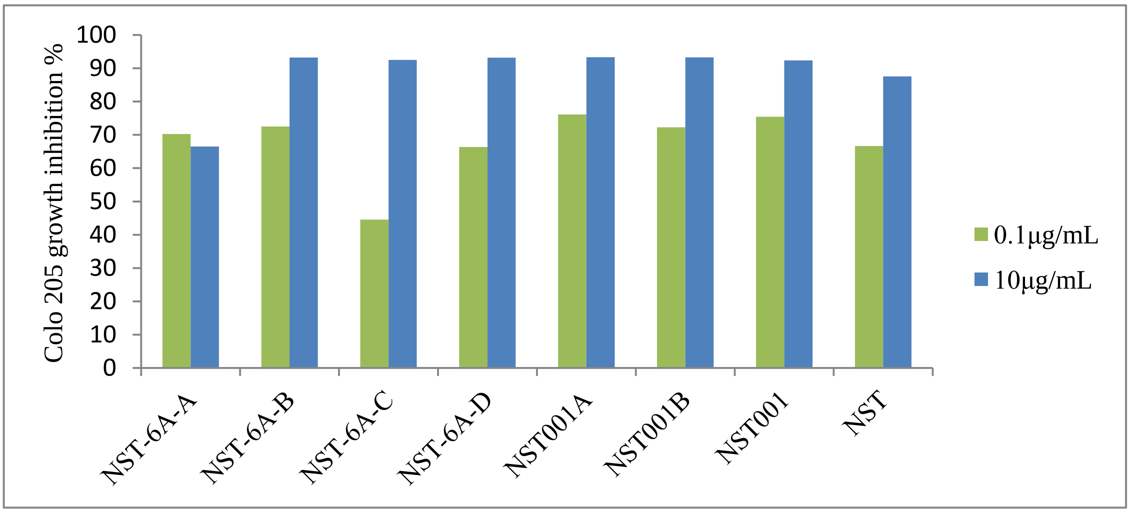

As **NST001** and **NST001A** showed most potent inhibition at both concentrations, these two compounds were selected to identify precise IC_{50} values on human non-small cell lung cell line H522, human colon cancer cell line Colo 205, human hepatocellular liver carcinoma cell line HepG2, human breast adenocarcinoma cell line MDA-MB-468 and human gastric cancer cell line BGC823. As shown in Figure S4, **NST001A** was more sensitive to all human cancer cells, especially Colo 205 cell. Therefore, **NST001A** was selected to conduct further *in vivo* assays.

A possible reaction mechanism for the synthesis of the intermediate compounds NST001 and NST6A~D was outlined in Scheme S1.

Scheme S1. Synthetic scheme of the designed compounds.

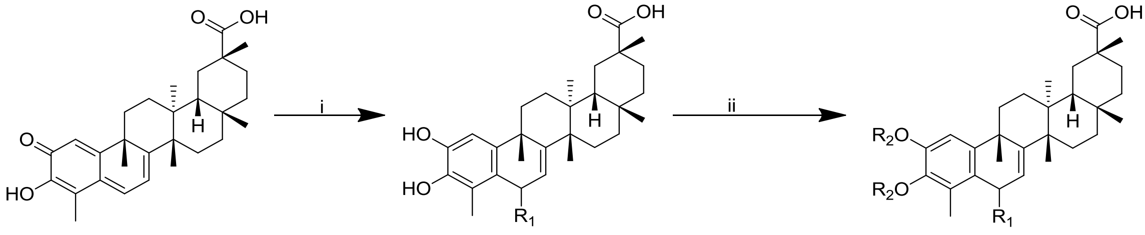

celastrol (**1**)

NST001 R_1 = $-SO_3Na$
NST6A R_1 = $-CH_2COCH_3$
NST6B R_1 = $-S(p\text{-}C_6H_4)Me$
NST6C R_1 = $-S(p\text{-}C_6H_4)NHCOCH_3$
NST6D R_1 = $-SCH_2CH(CO_2H)NHCOCH_3$

NST001A R_1 = $-SO_3Na$, R_2 = $-COCH_3$
NST001B R_1 = $-SO_3Na$, R_2 = $-COCH_2CH_3$
NST6A-A R_1 = $-CH_2COCH_3$, R_2 = $-COCH_3$
NST6A-B R_1 = $-S(p\text{-}C_6H_4)Me$, R_2 = $-COCH_3$
NST6A-C R_1 = $-S(p\text{-}C_6H_4)NHCOCH_3$, R_2 = $-COCH_3$
NST6A-D R_1 = $-SCH_2CH(CO_2H)NHCOCH_3$, R_2 = $-COCH_3$

Reagents and conditions: (i) For **NST001**: $NaHSO_3$, MeOH, RT, 1.5 h, N_2; For **NST6A**: acetone, 1 N HCl (cat.), RT, N_2; For **NST6A~D**: RSH, MeOH, RT, N_2; (ii) Ac_2O, Py, RT, N_2; or $(CH_3CH_2CO)_2O$, Py, RT, N_2.

Possible mechanism of the synthesis of NST001and NST6A~D:

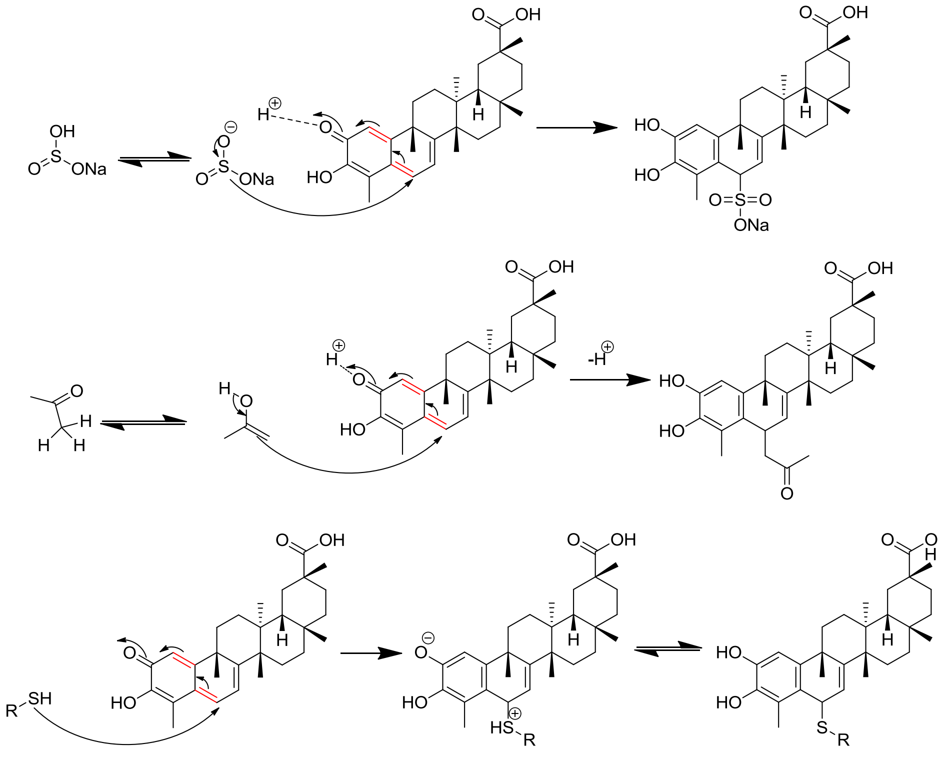

Table S2. The change of the mice body weight and the inhibitory rate of tumor(T/C) of different drug loaded system against Colo 205 tumor in nude mice (mean ±SD).

Group	N.O. of Mice		Body Weight (g)		Tumer Volume (TV, mm^3)		T/C/%	Tumer Weight (g)	IRT/%
	Initial	Final	Initial	Final	Initial	Final			
model	12	12	20.50 ±1.45	19.83 ±2.08	118.13 ±36.46	1528.59 ±415.00	--	0.99 ±0.25	--
Cisplatin (4mg/kg)	7	7	20.71 ±1.50	15.57 ±1.40 ***	122.11 ±47.85	793.58 ±379.56 **	49.32	0.45 ±0.25 ***	55.00
NST001A (6mg/kg)	6	6	20.33 ±1.03	18.83 ±2.79	119.97 ±27.66	910.83 ±301.60 **	56.75	0.55 ±0.23 **	44.66
NST001A (12mg/kg)	6	6	21.33 ±0.82	18.00 ±2.19	114.01 ±26.15	1104.71 ±494.57	77.25	0.68 ±0.35 *	31.82
NST001A (24mg/kg)	6	6	21.33 ±0.52	18.33 ±1.21	118.05 ±39.37	818.65 ±162.53 **	54.00	0.49 ±0.09 ***	50.96

All date are means ±SD. --: Not detect, * $p < 0.05$, ** $p < 0.01$, *** $p < 0.001$ when compared with model.

Figure S7. Effects of NST001A on the growth of Colo 205 xenografts in nude mice.

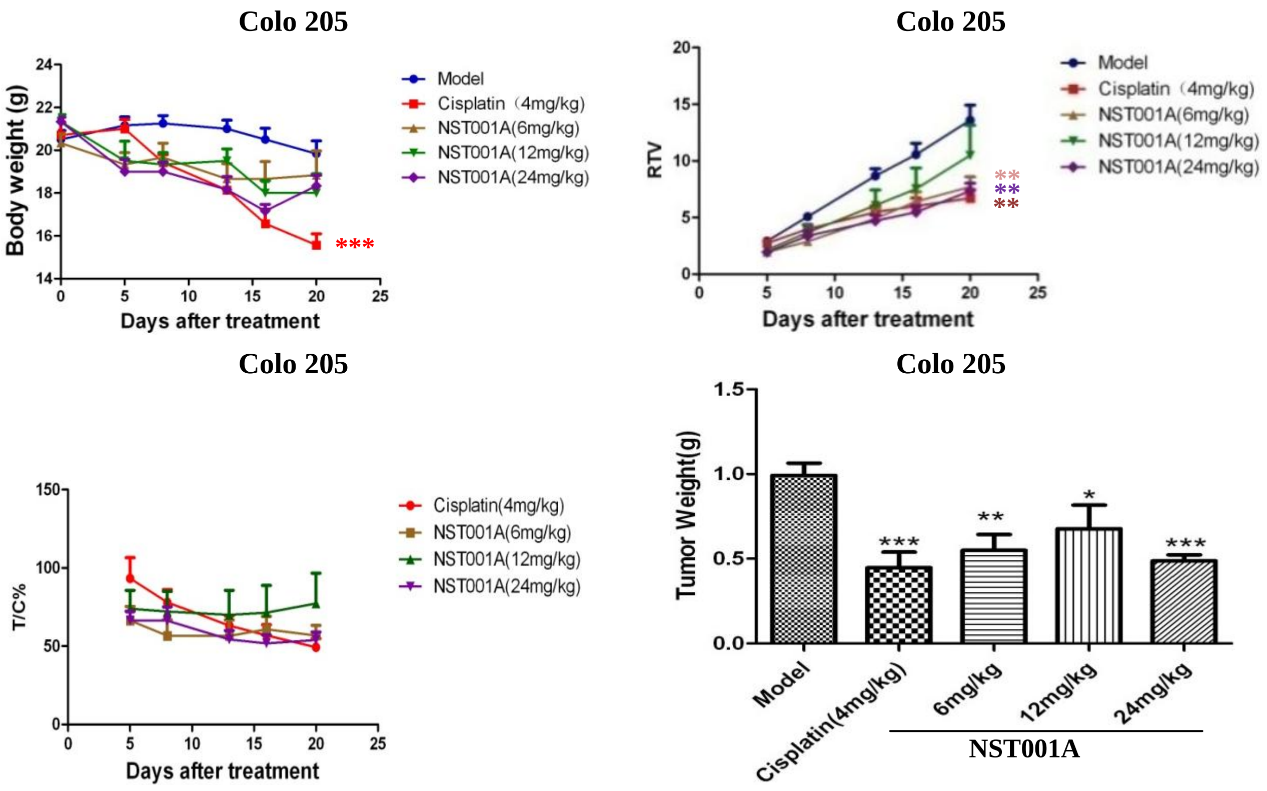

All date are means ±SD. * $p < 0.05$, ** $p < 0.01$, *** $p < 0.001$ when compared with model.

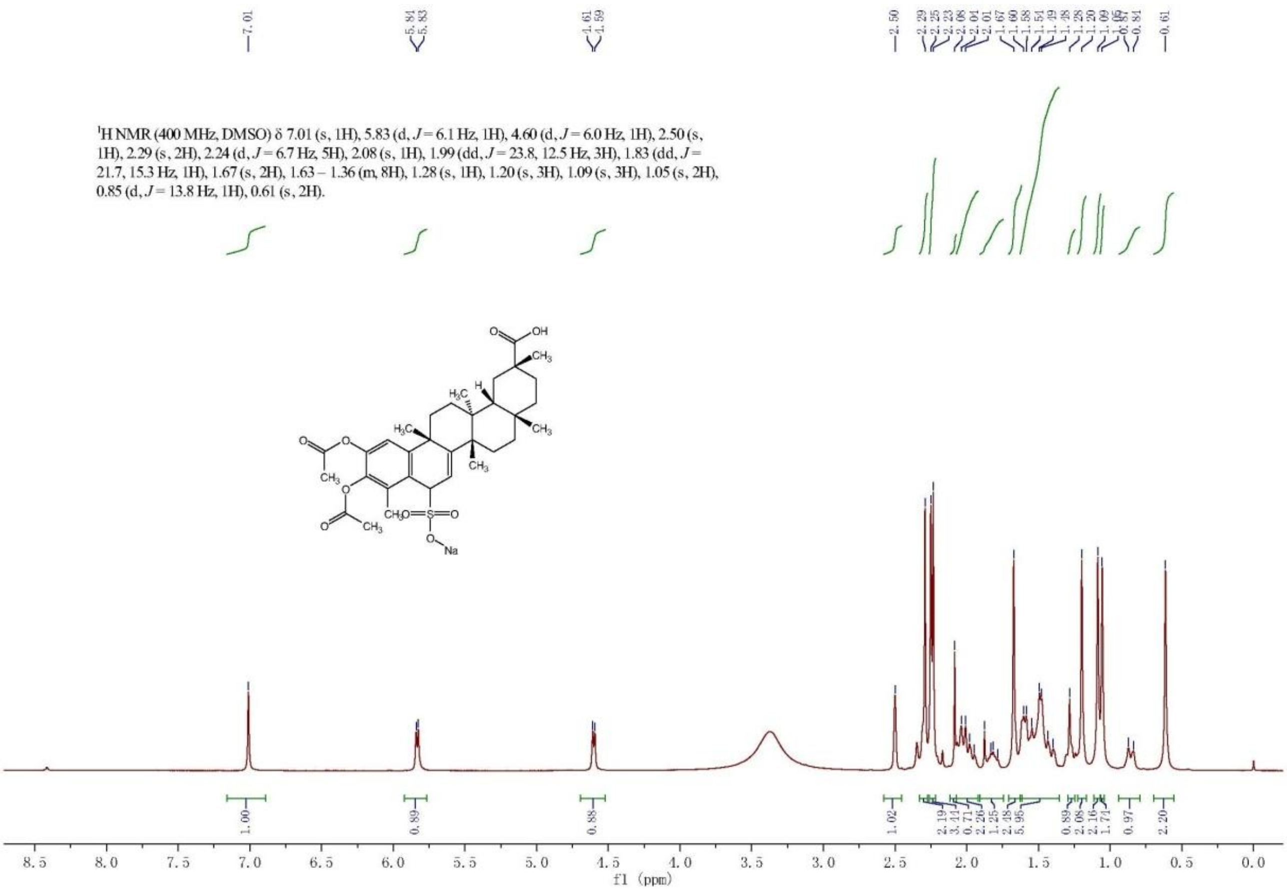

^{1}H-NMR (NST001A)

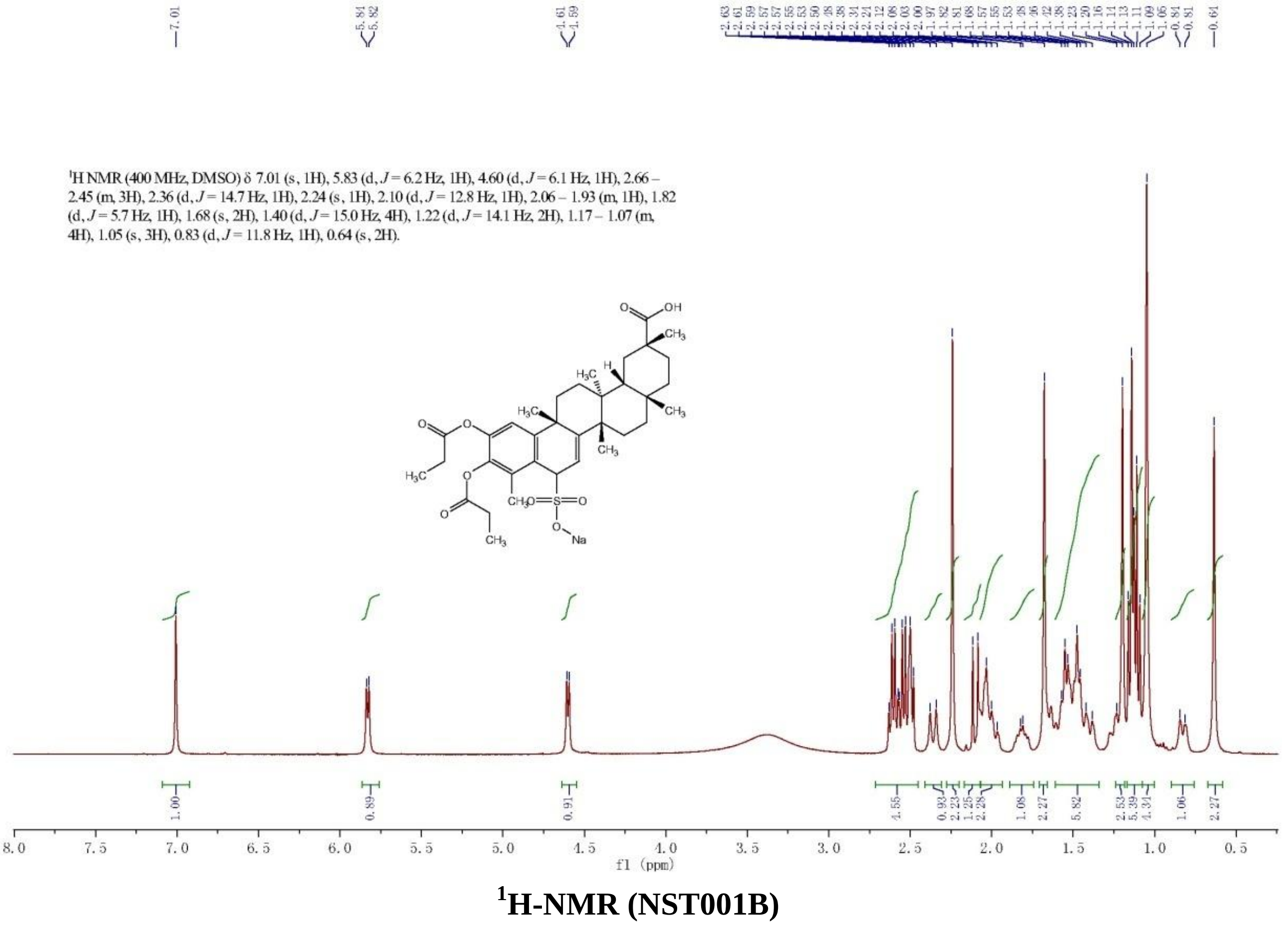

^{1}H-NMR (NST001B)

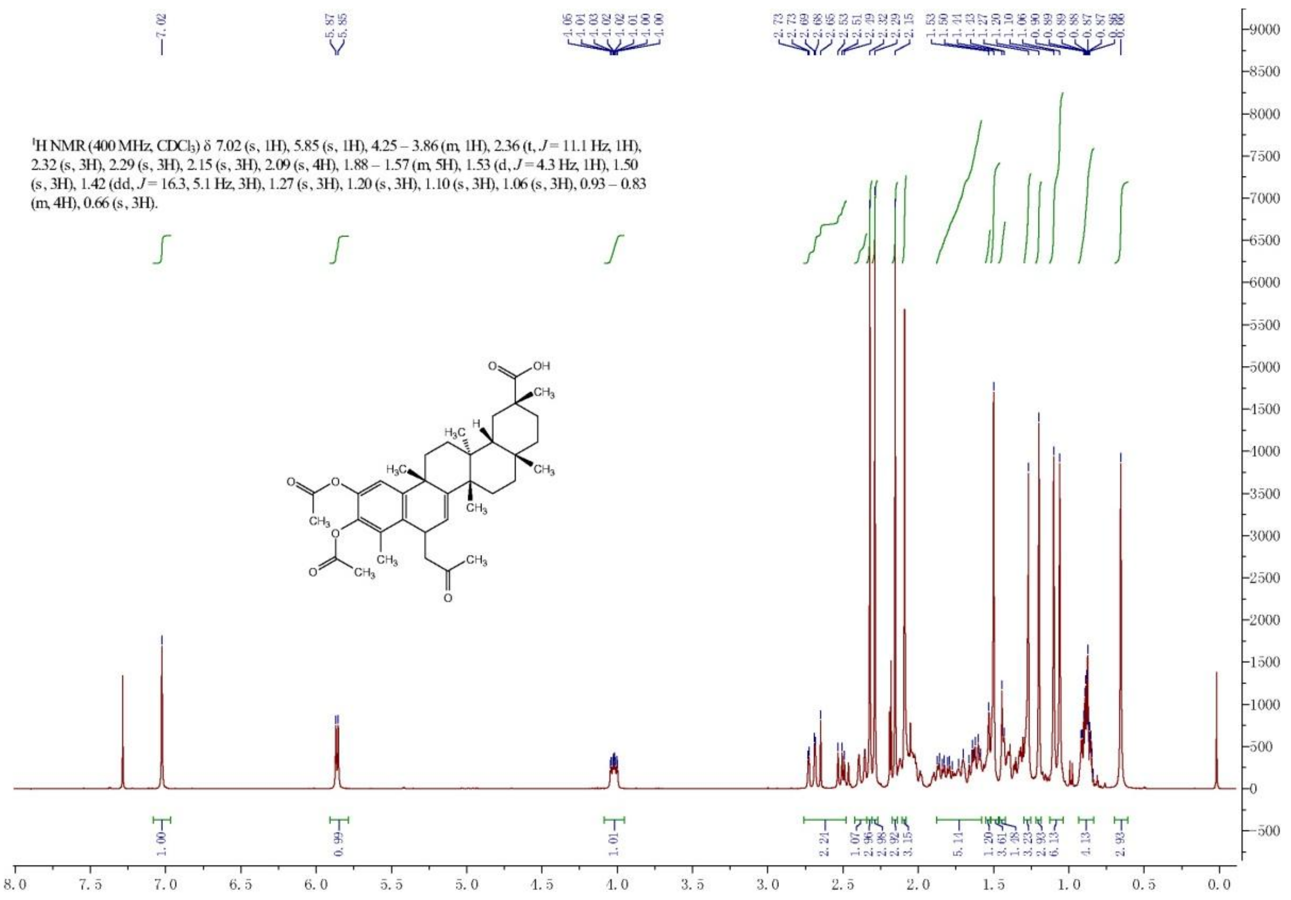

^{1}H-NMR (NST6A-A)

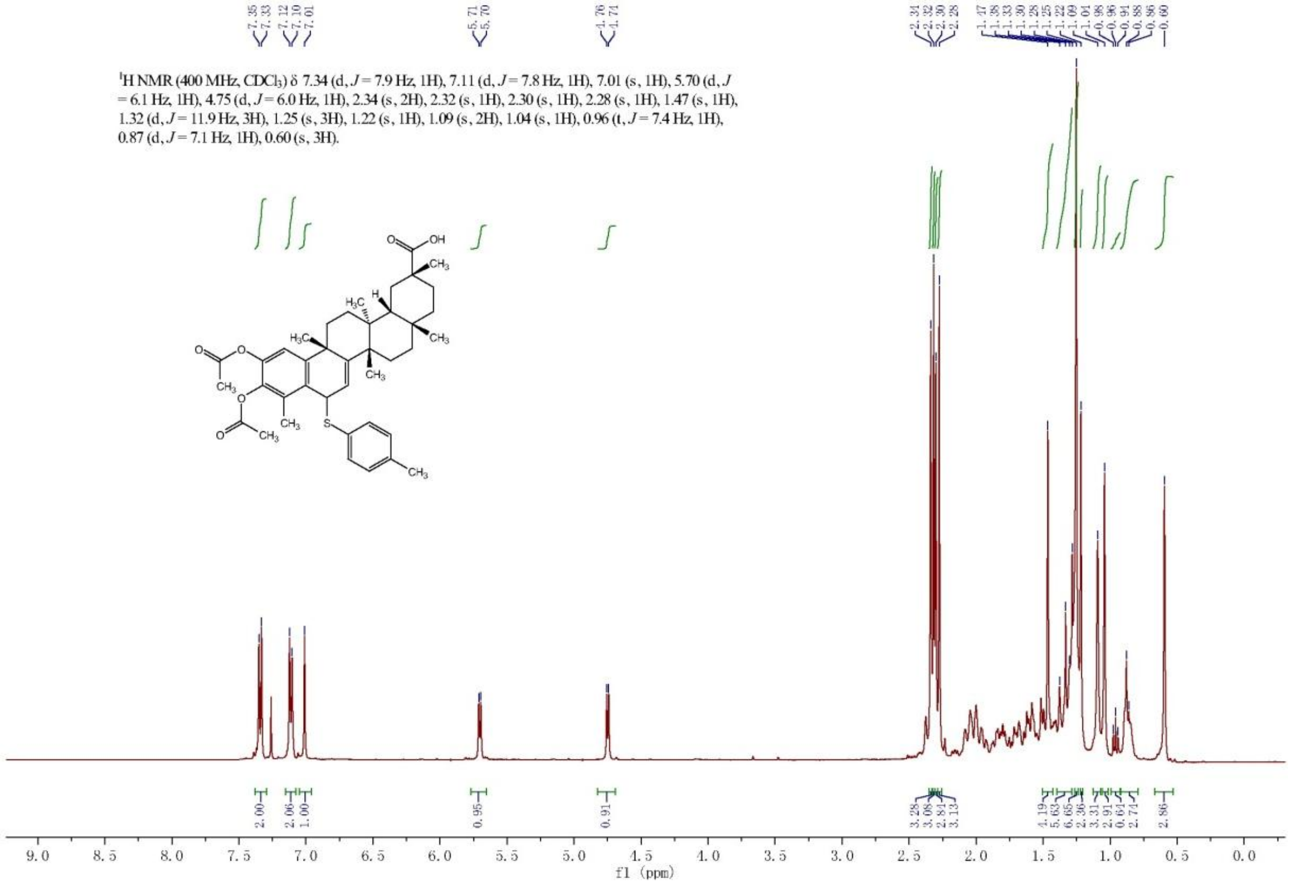

^{1}H-NMR (NST6A-B)

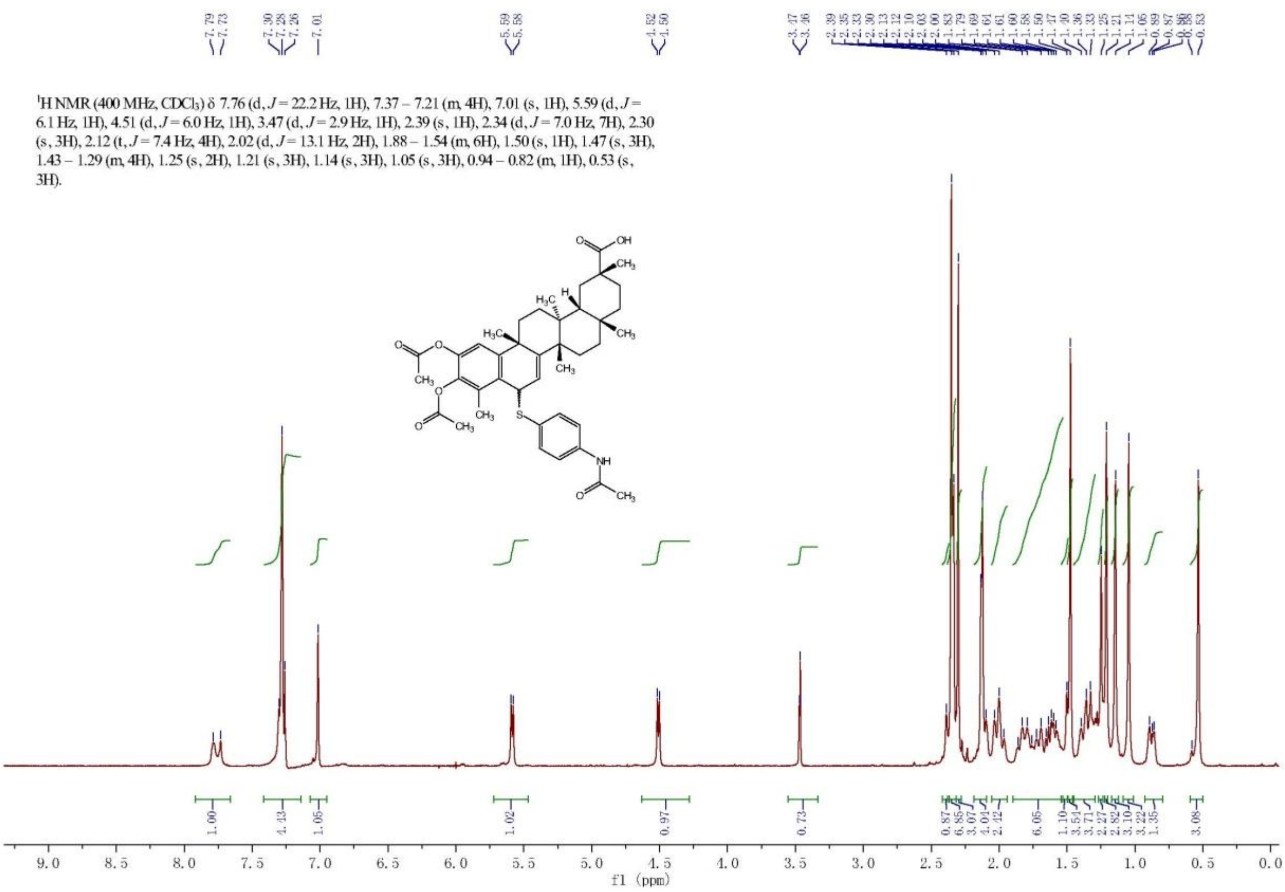

^{1}H-NMR (NST6A-C)

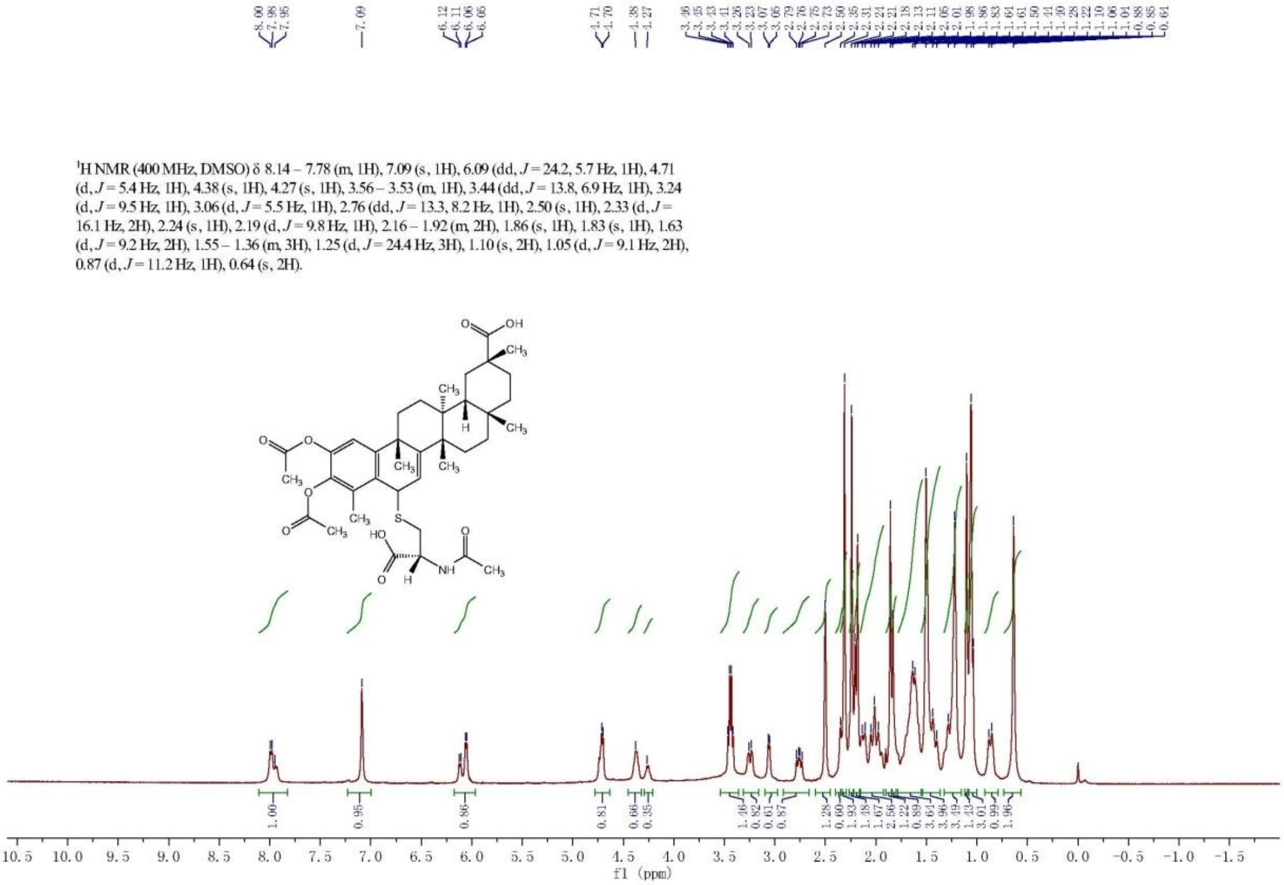

^{1}H-NMR (NST6A-D)

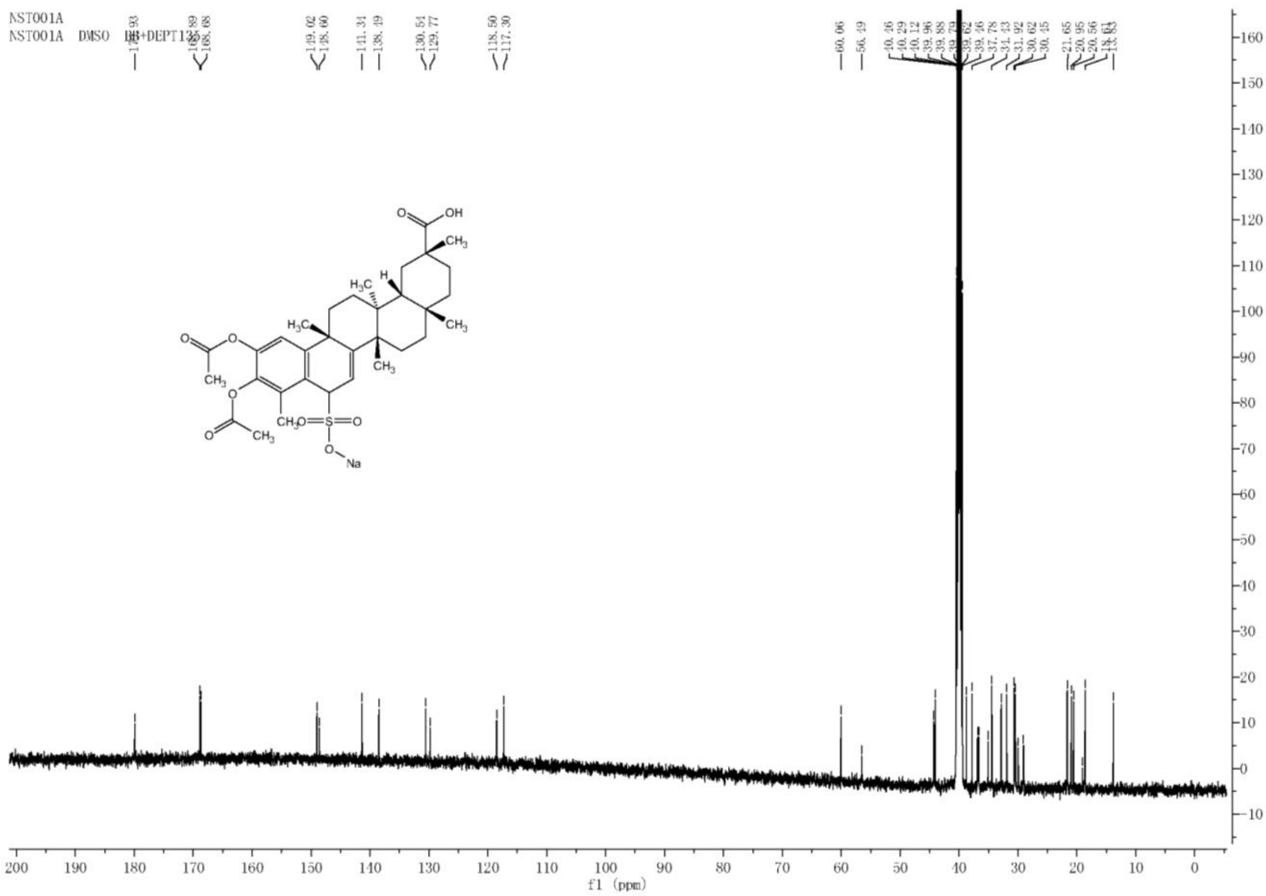

^{13}C-NMR (NST001A)

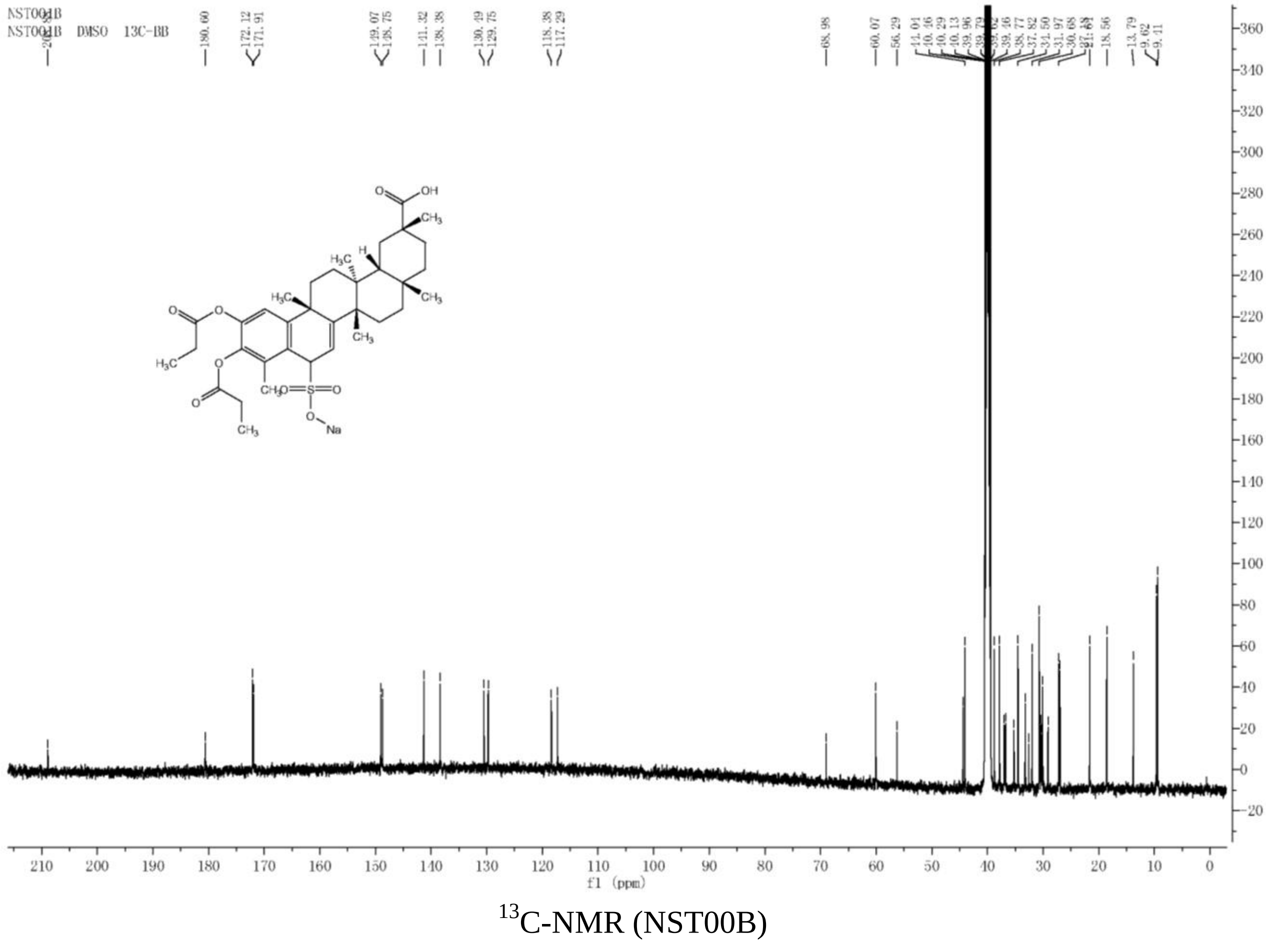

^{13}C-NMR (NST00B)

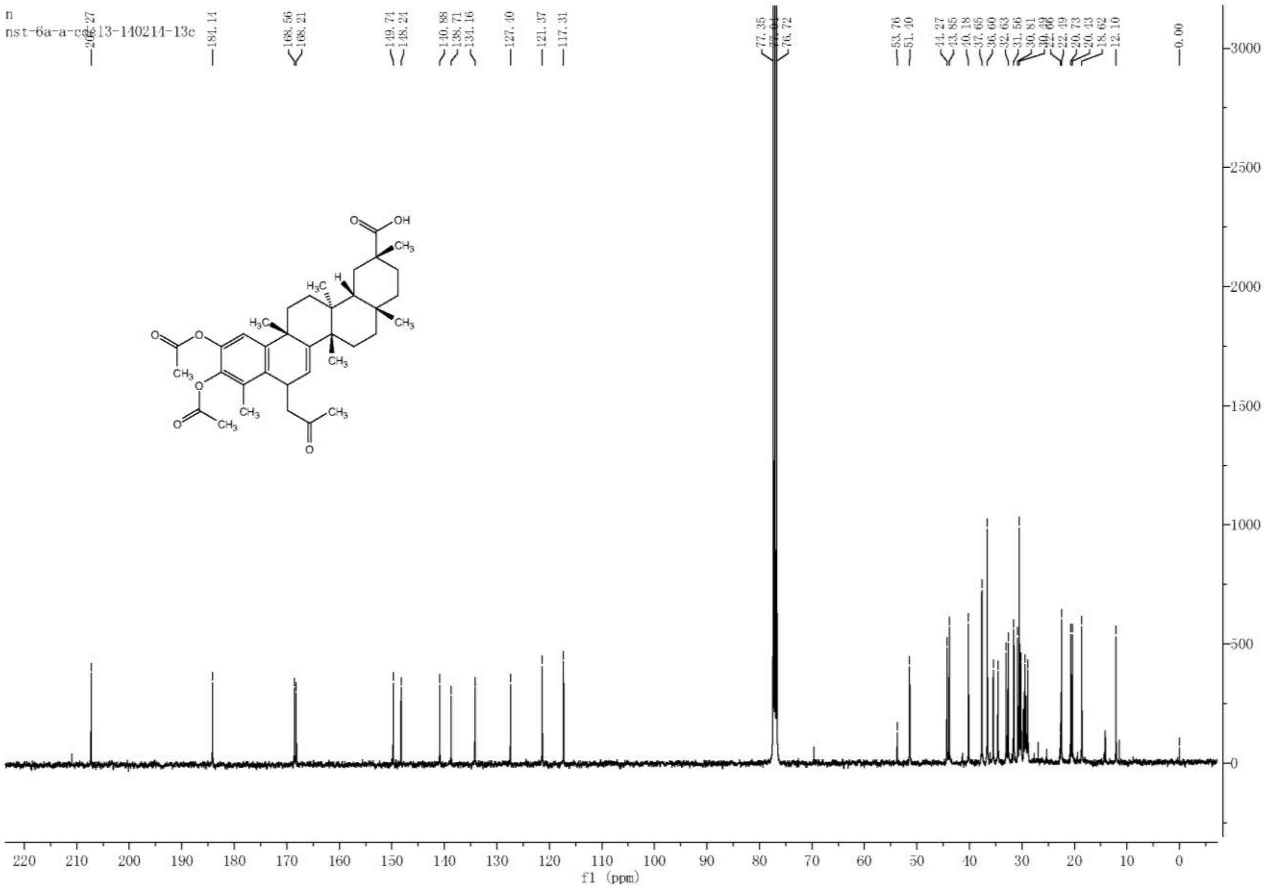

^{13}C-NMR (NST6A-A)

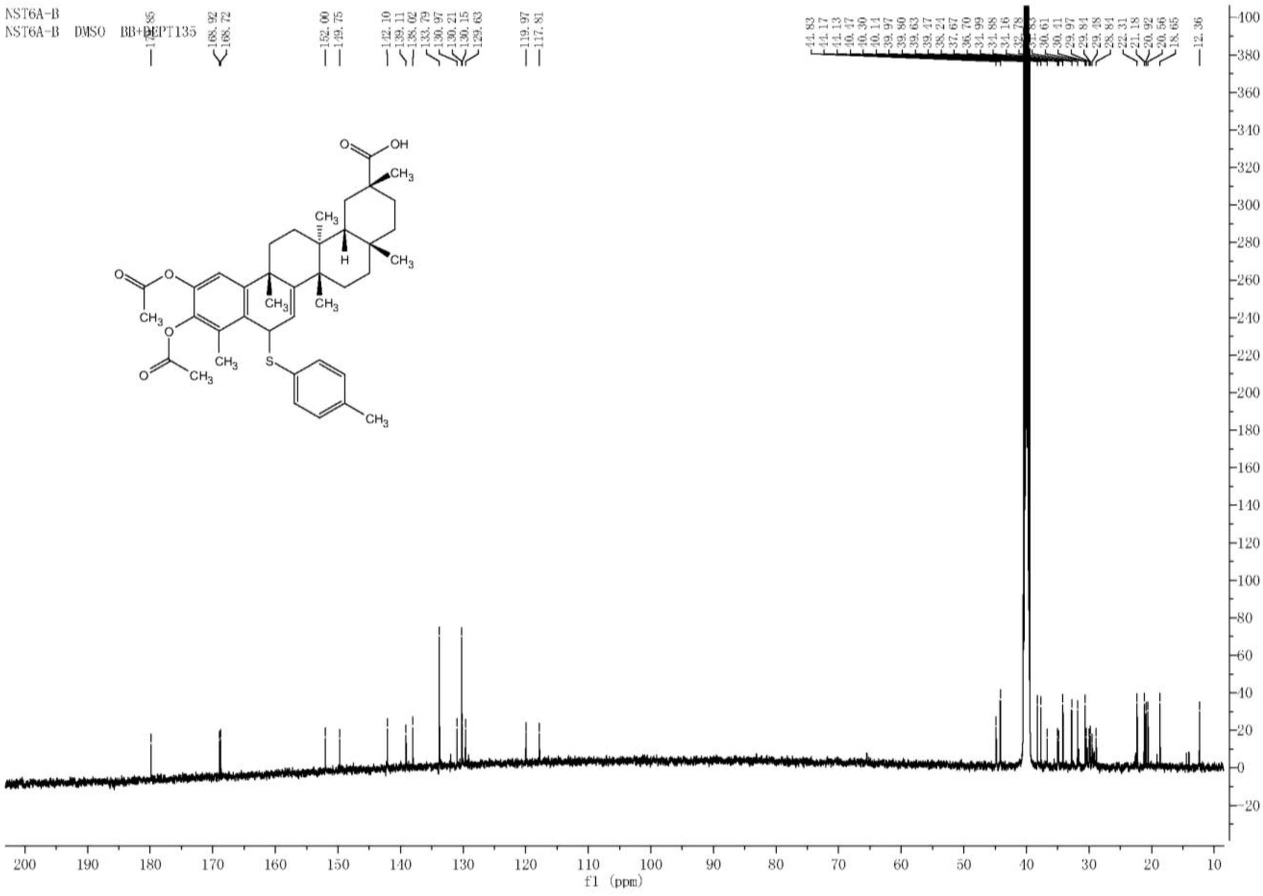

^{13}C-NMR (NST6A-B)

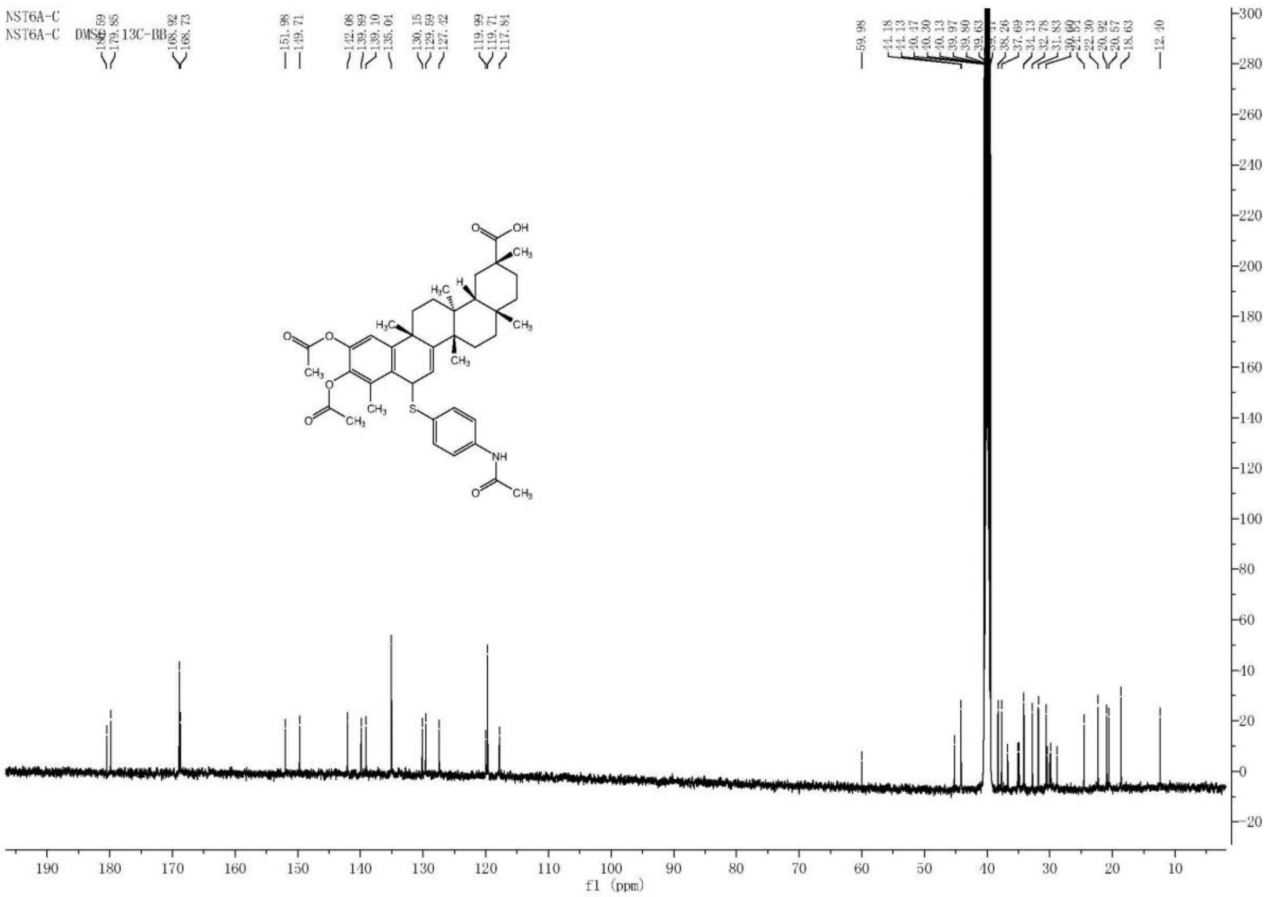

^{13}C-NMR (NST6A-C)

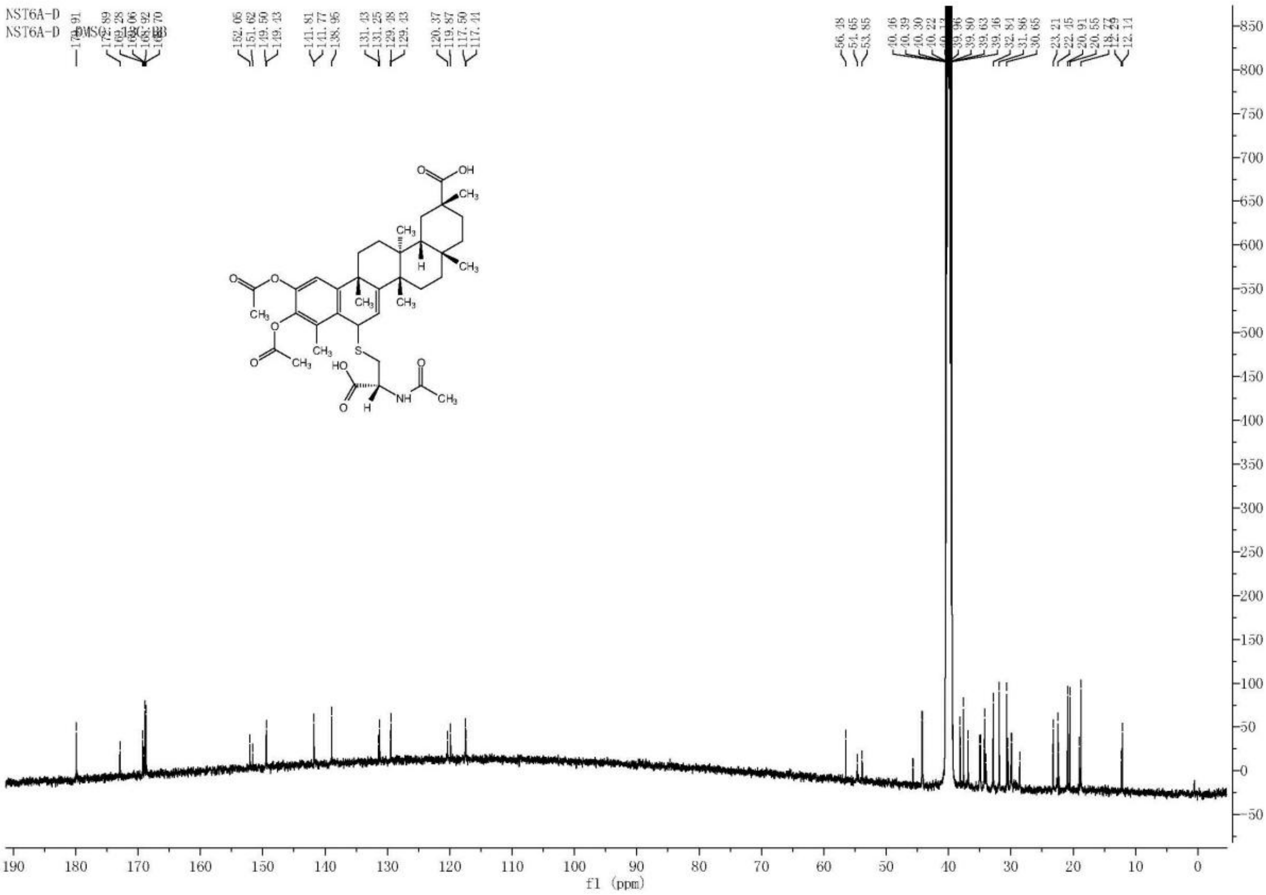

^{13}C-NMR (NST6A-D)

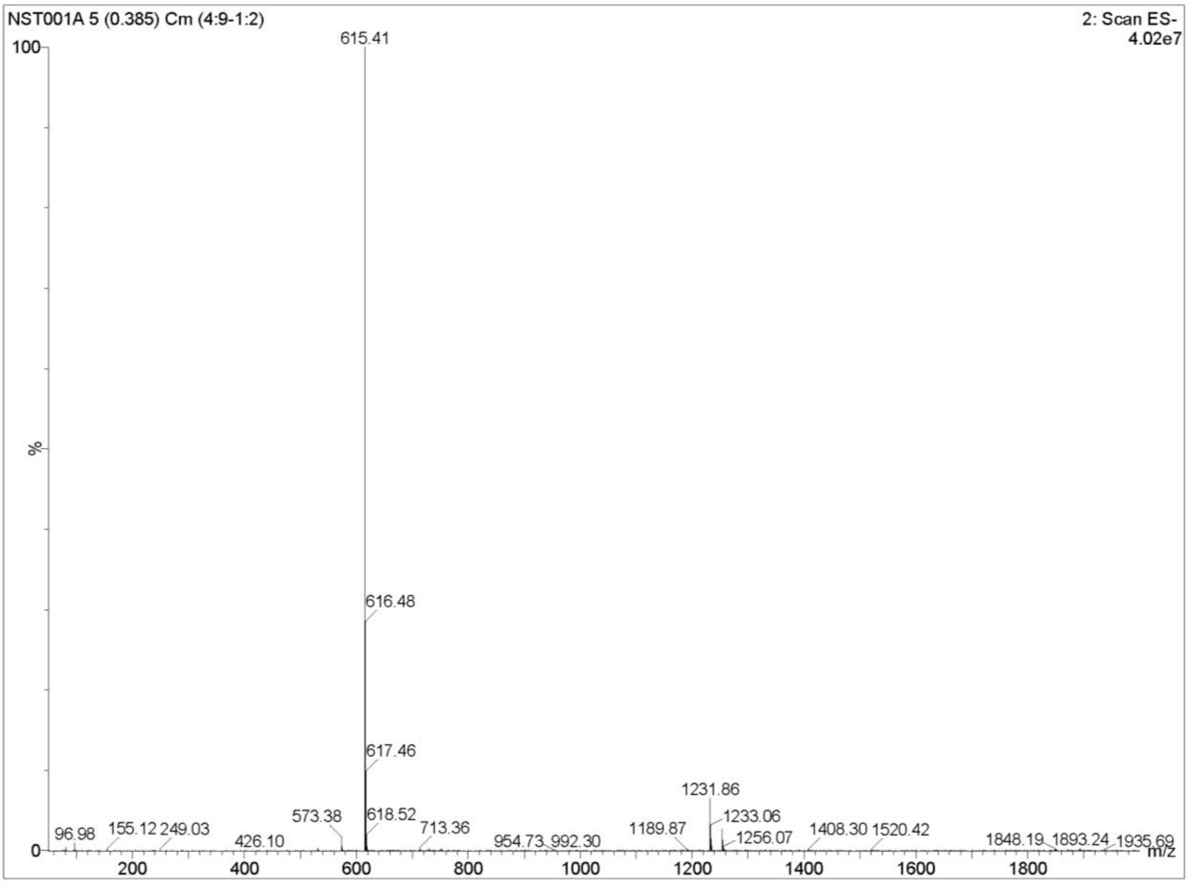

LC-ESI-MS(−) (NST001A)

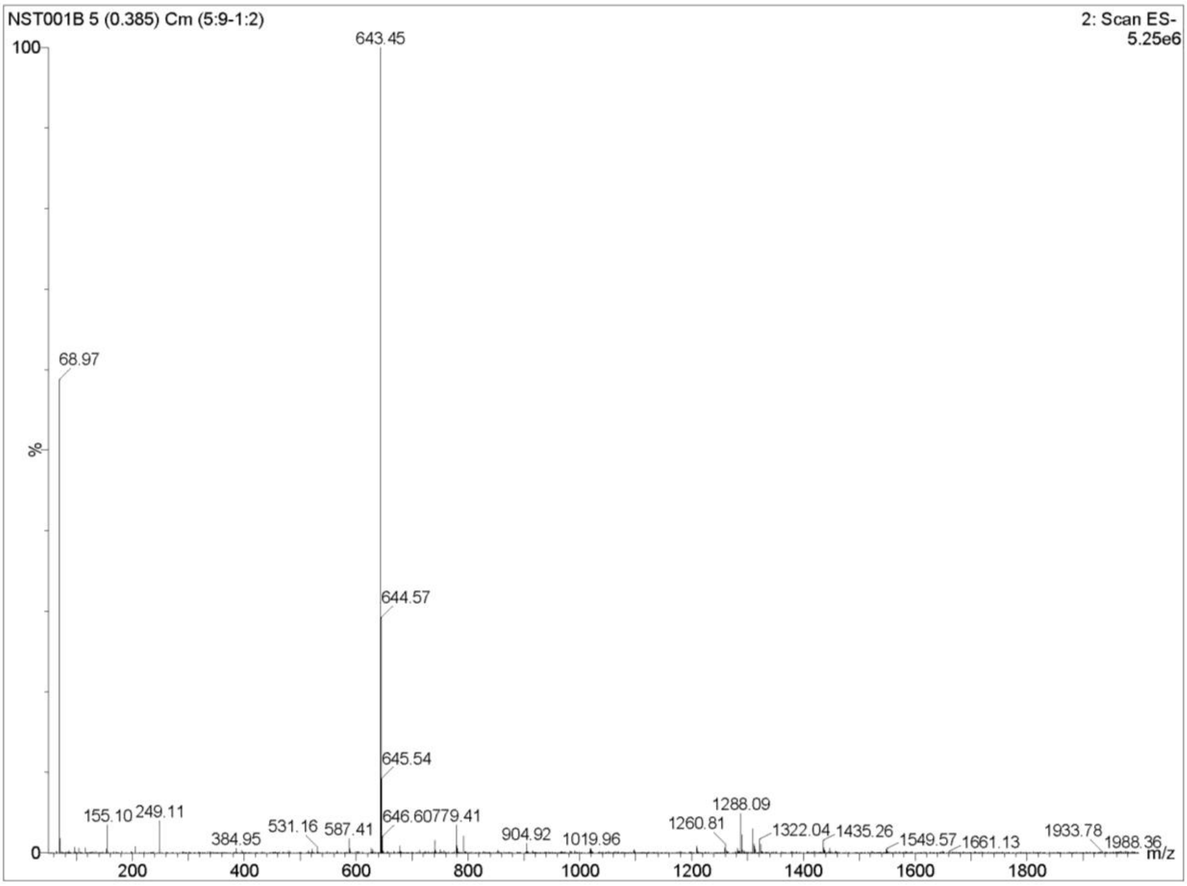

LC-ESI-MS(−) (NST001B)

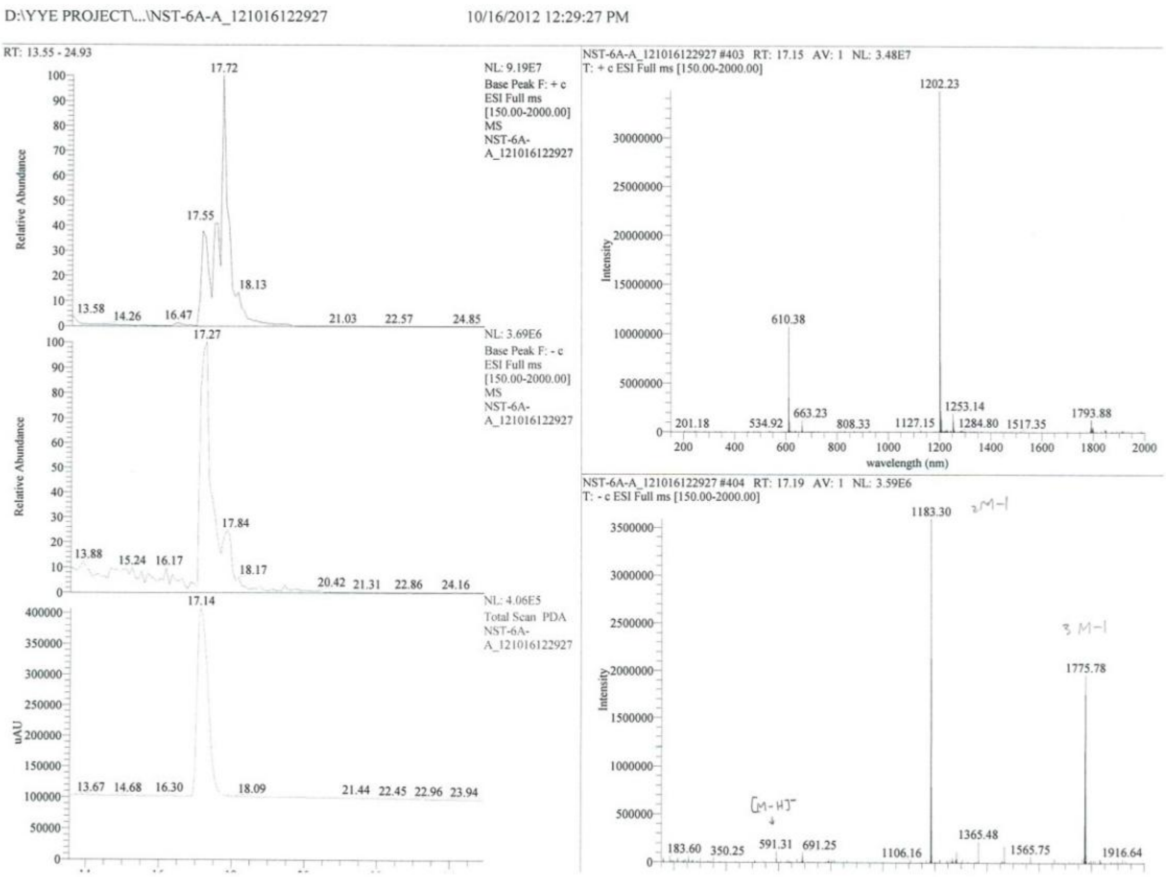

LC-ESI-MS(−) (NST6A-A)

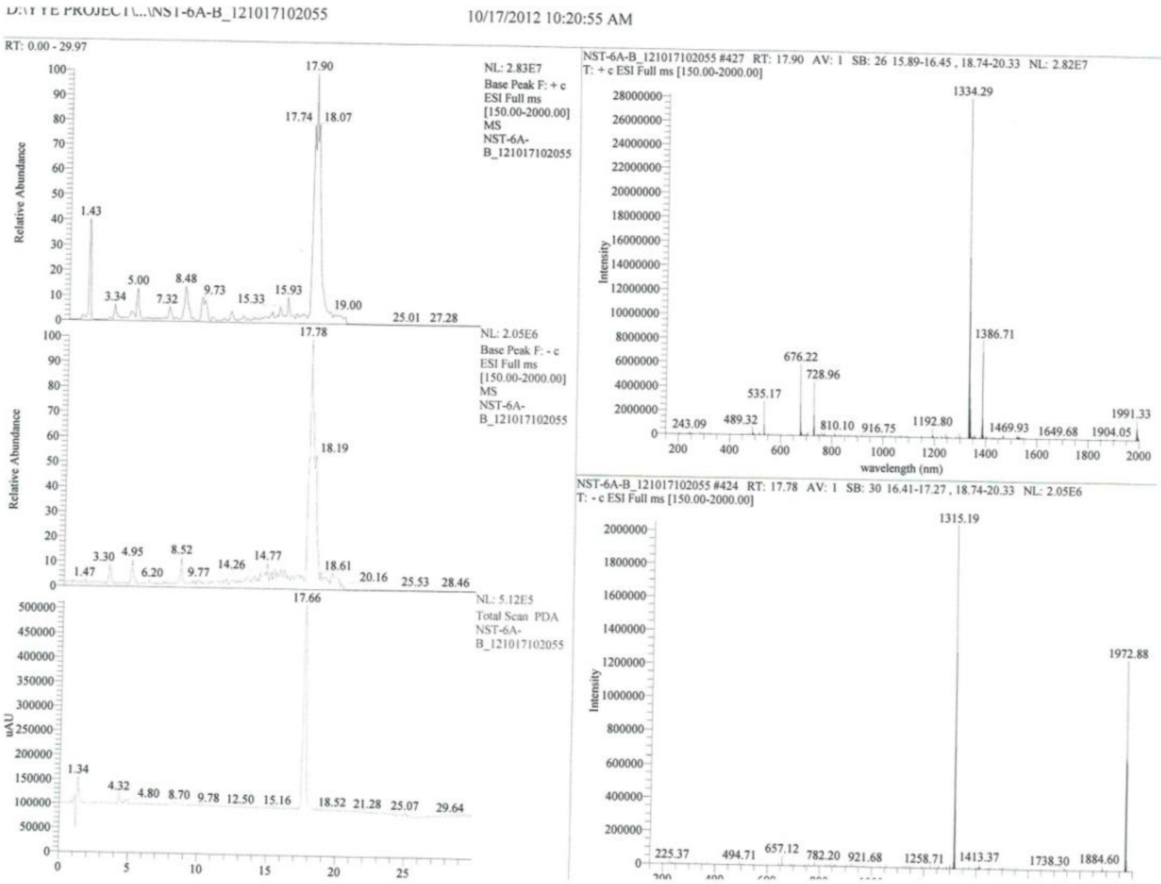

LC-ESI-MS(−) (NST6A-B)

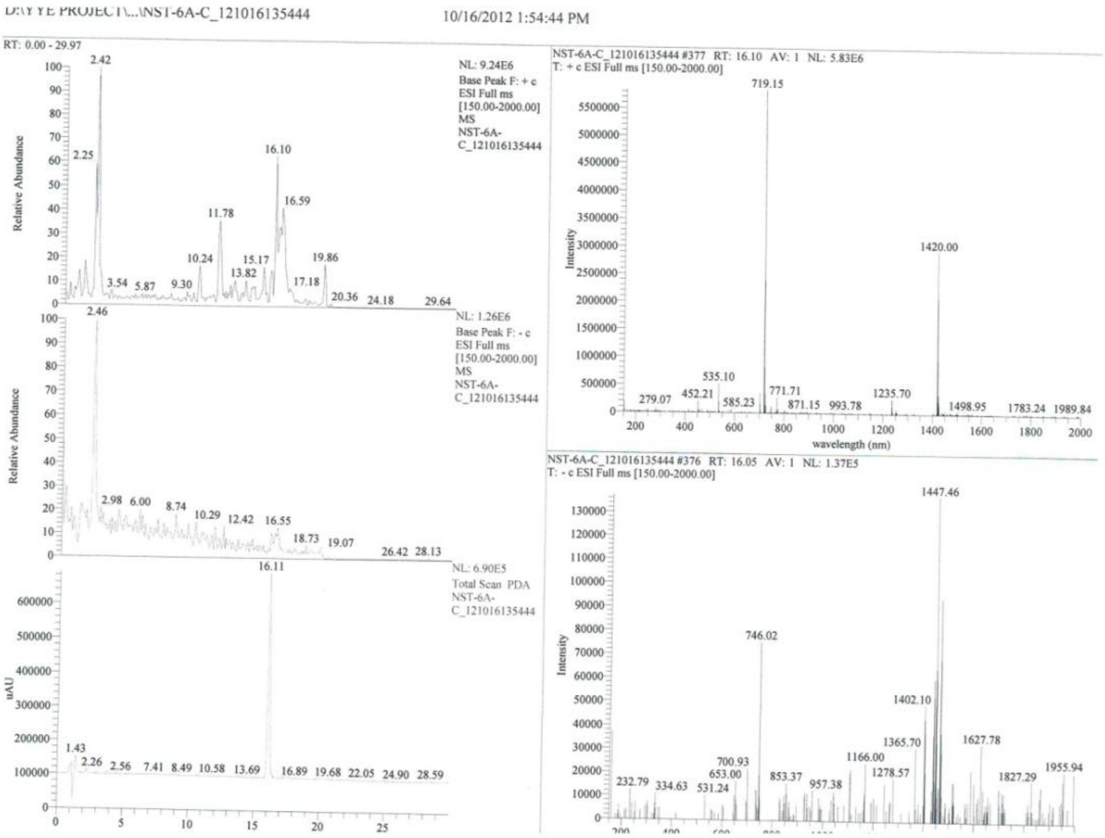

LC-ESI-MS(−) (NST6A-C)

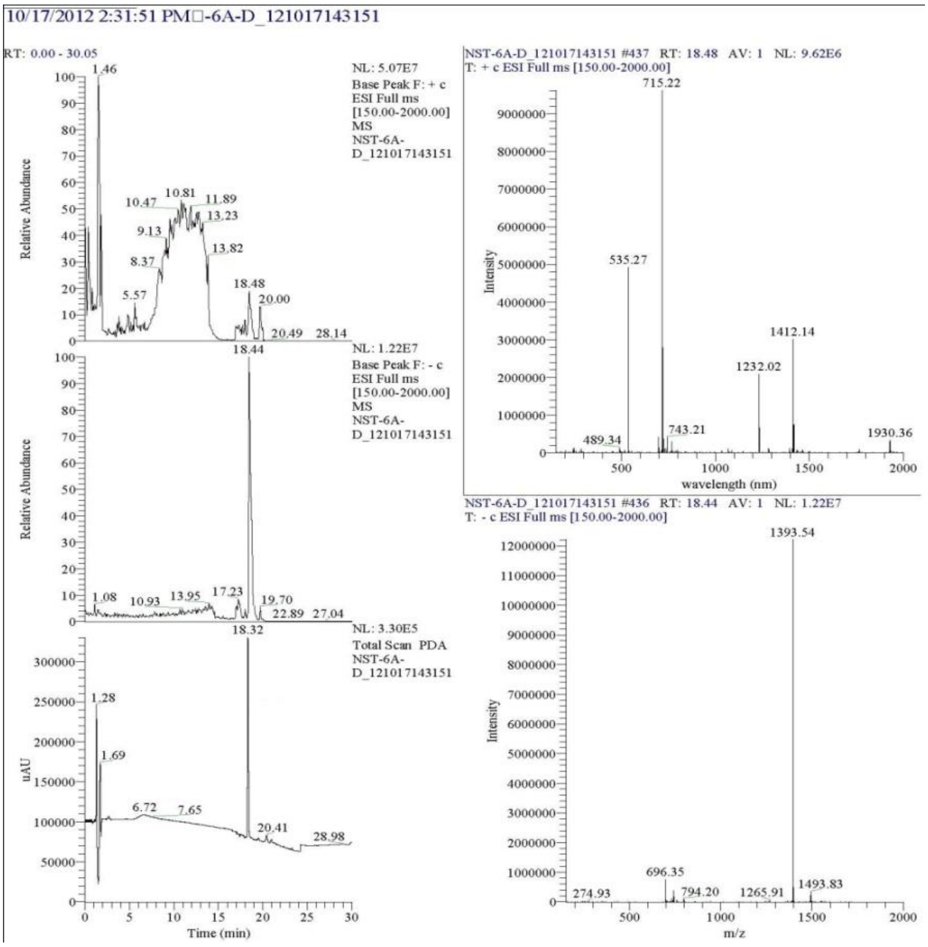

LC-ESI-MS(−) (NST6A-D)

SHIMADZU LabSolutions

Analysis Report

<Sample Information>

Sample Name	: NST001		
Data Filename	: NST001		
Method Filename	: 9-NC.lcm		
Vial #	:		
Injection Volume	: 20 uL	Level	: 1
Date Acquired	: 6/8/2012 5:16:04 AM	Acquired by	: Administrator
Date Processed	: 6/16/2014 3:33:03 PM	Processed by	: System Administrator

<Chromatogram>

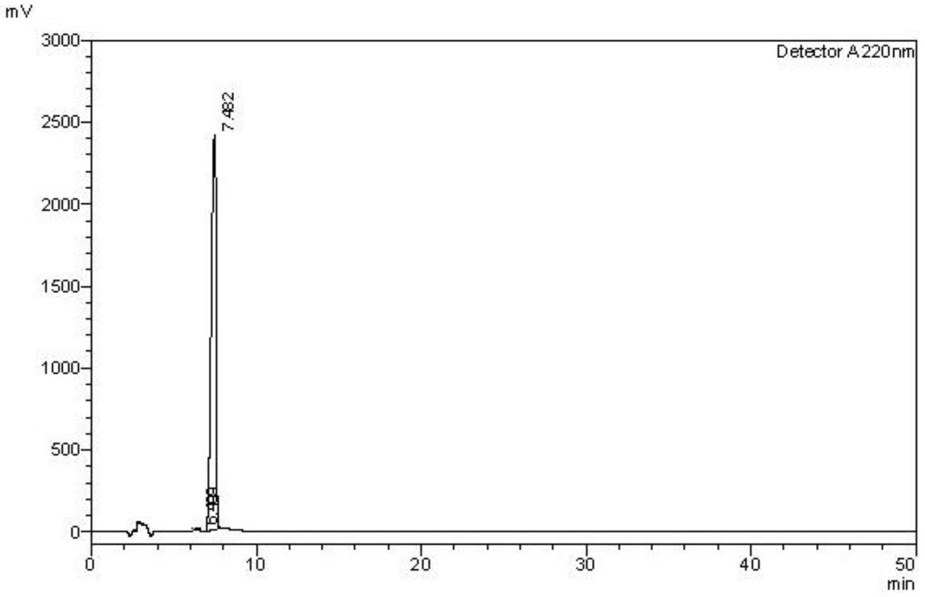

<Peak Table>

Detector A 220nm

Peak#	Ret. Time	Area	Area%	Height	tical Plates/mete
1	6.499	106181	0.213	10193	65320
2	7.482	49678504	99.787	2410630	20932
Total		49784685	100.000	2420823	

D:\220nm\NST001.lcd

HPLC purity (NST001)

SHIMADZU LabSolutions Analysis Report

<Sample Information>

Sample Name	: NST001A		
Data Filename	: NST001A		
Method Filename	: CV.lcm		
Vial #	:		
Injection Volume	: 20 uL	Level	: 1
Date Acquired	: 3/12/2012 9:55:35 PM	Acquired by	: Administrator
Date Processed	: 6/16/2014 2:57:27 PM	Processed by	: System Administrator

<Chromatogram>

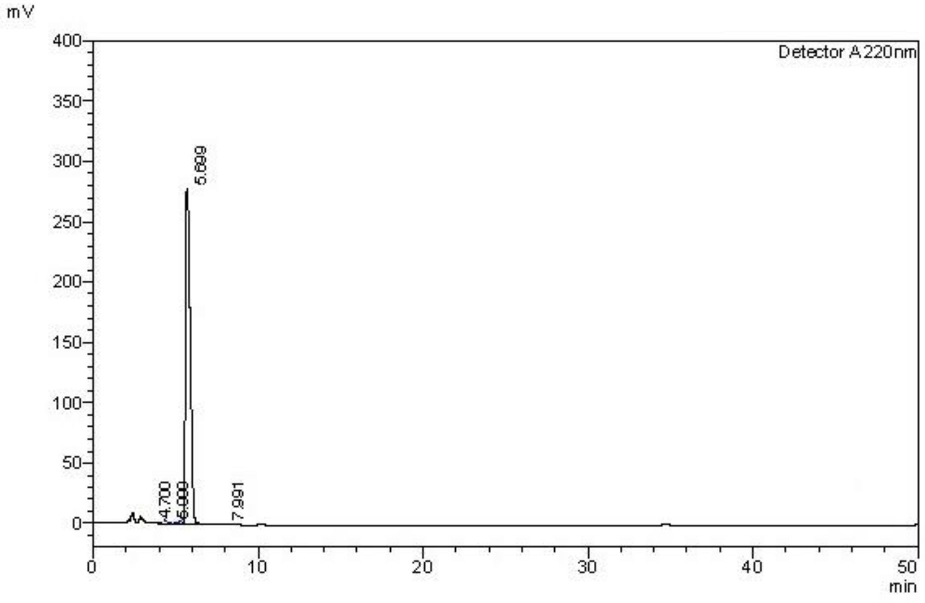

<Peak Table>

Detector A 220nm

Peak#	Ret. Time	Area	Area%	Height	tical Plates/mete
1	4.700	5808	0.104	809	56660
2	5.000	4310	0.077	674	80269
3	5.699	5588448	99.742	278144	12420
4	7.991	4354	0.078	483	98610
Total		5602919	100.000	280109	

HPLC purity (NST001A)

6/16/2014 3:13:53 PM Page 1 / 1

SHIMADZU LabSolutions **Analysis Report**

<Sample Information>

Sample Name : NST001B
Data Filename : NST001B
Method Filename : NST analog.lcm
Vial # :
Injection Volume : 20 uL
Date Acquired : 6/12/2014 8:56:17 PM
Date Processed : 6/15/2014 11:01:04 PM
Level : 1
Acquired by : Administrator
Processed by : Administrator

<Chromatogram>

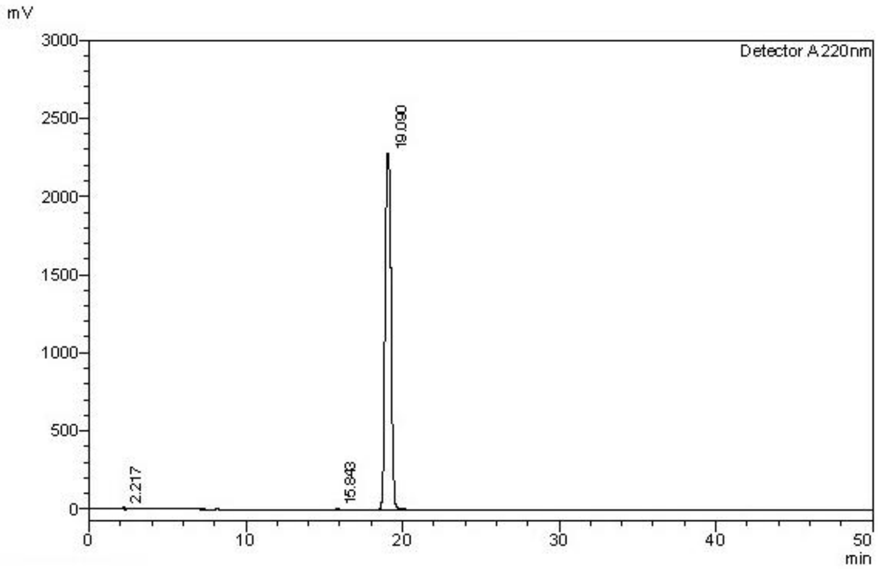

<Peak Table>

Detector A 220nm

Peak#	Ret. Time	Area	Area%	Height	tical Plates/mete
1	2.217	20950	0.036	14049	27390
2	15.843	51080	0.087	3036	126111
3	19.090	58596749	99.877	2280134	99228
Total		58668780	100.000	2297220	

G:\220nm\NST14.lcd

HPLC purity (NST001B)

SHIMADZU LabSolutions

Analysis Report

<Sample Information>

Sample Name	: NST6A-A		
Data Filename	: NST6A-A		
Method Filename	: NST analog.lcm		
Vial #	:		
Injection Volume	: 20 uL	Level	: 1
Date Acquired	: 6/13/2014 1:26:24 AM	Acquired by	: Administrator
Date Processed	: 6/15/2014 11:06:32 PM	Processed by	: Administrator

<Chromatogram>

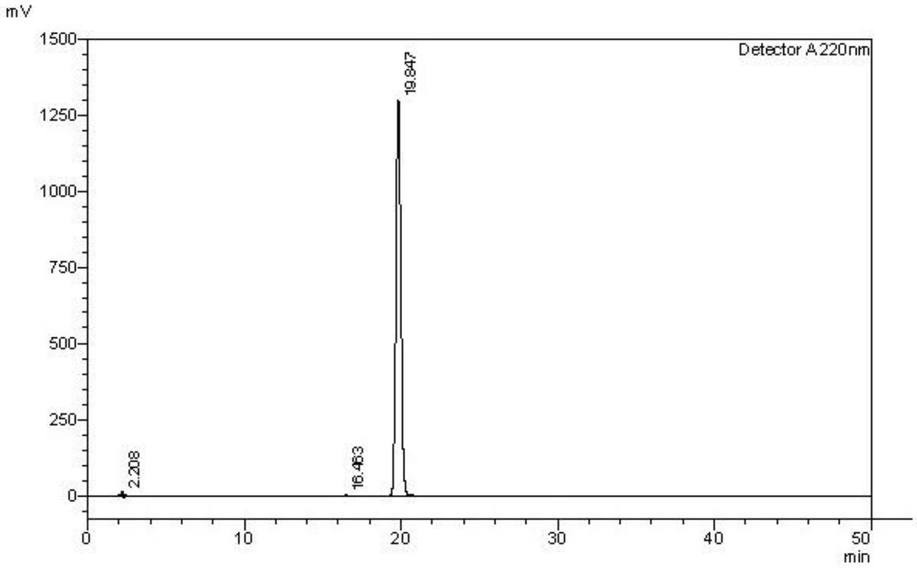

<Peak Table>

Detector A 220nm

Peak#	Ret. Time	Area	Area%	Height	tical Plates/mete
1	2.208	48158	0.168	13138	22329
2	16.463	21454	0.075	1241	132149
3	19.847	28523582	99.757	1296761	124165
Total		28593195	100.000	1311140	

HPLC purity (NST6A-A)

SHIMADZU LabSolutions

Analysis Report

<Sample Information>

Sample Name : NST6A-B
Data Filename : NST6A-B
Method Filename : NST analog.lcm
Vial # :
Injection Volume : 20 uL
Date Acquired : 6/12/2014 11:02:15 PM
Date Processed : 6/15/2014 11:03:16 PM
Level : 1
Acquired by : Administrator
Processed by : Administrator

<Chromatogram>

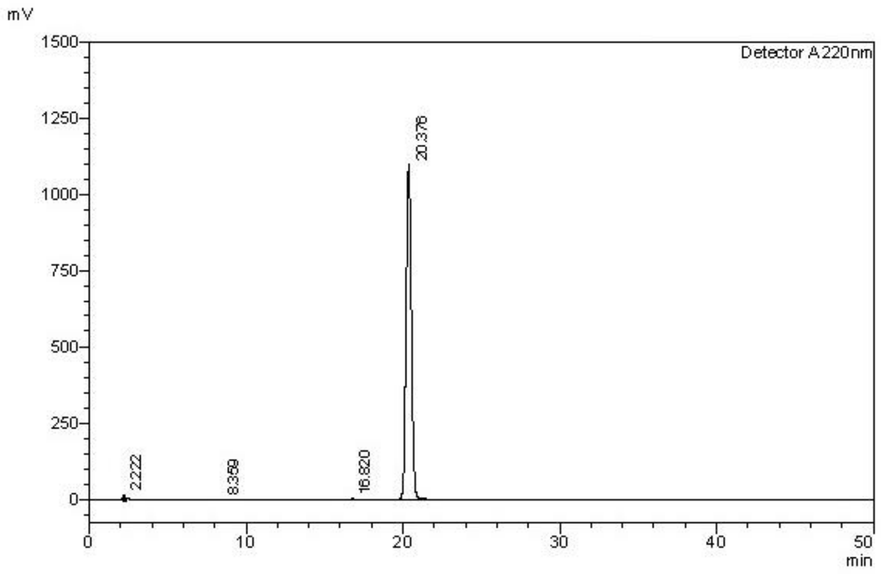

<Peak Table>

Detector A 220nm

Peak#	Ret. Time	Area	Area%	Height	tical Plates/mete
1	2.222	126424	0.498	19608	19755
2	8.359	6567	0.026	555	90775
3	16.820	18350	0.072	1041	136576
4	20.376	25238468	99.404	1099030	120472
Total		25389807	100.000	1120233	

HPLC purity (NST6A-B)

SHIMADZU LabSolutions Analysis Report

<Sample Information>

Sample Name : NST6A-C
Data Filename : NST6A-C
Method Filename : NST analog.lcm
Vial # :
Injection Volume : 20 uL
Date Acquired : 6/13/2014 12:16:45 AM
Date Processed : 6/15/2014 11:06:20 PM
Level : 1
Acquired by : Administrator
Processed by : Administrator

<Chromatogram>

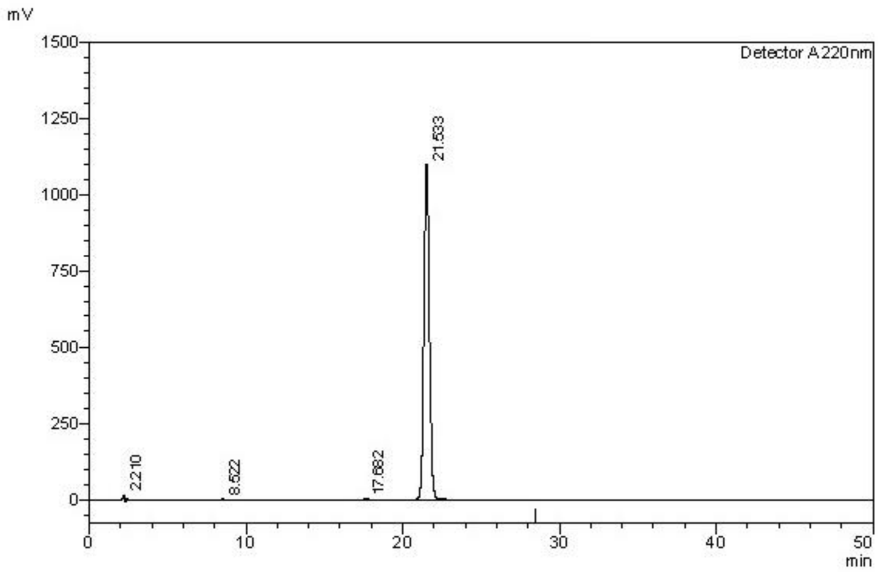

<Peak Table>

Detector A 220nm

Peak#	Ret. Time	Area	Area%	Height	tical Plates/mete
1	2.210	41428	0.155	13704	24148
2	8.522	7558	0.028	713	86112
3	17.682	19648	0.073	1065	142391
4	21.533	26734204	99.744	1097037	119743
Total		26802838	100.000	1112520	

HPLC purity (NST6A-C)

6/16/2014 3:24:34 PM Page 1 / 1

Analysis Report

<Sample Information>

Sample Name : NST6A-D
Data Filename : NST6A-D
Method Filename : NST analog.lcm
Vial # :
Injection Volume : 20 uL
Date Acquired : 6/12/2014 10:04:20 PM
Date Processed : 6/16/2014 3:19:27 PM
Level : 1
Acquired by : Administrator
Processed by : System Administrator

<Chromatogram>

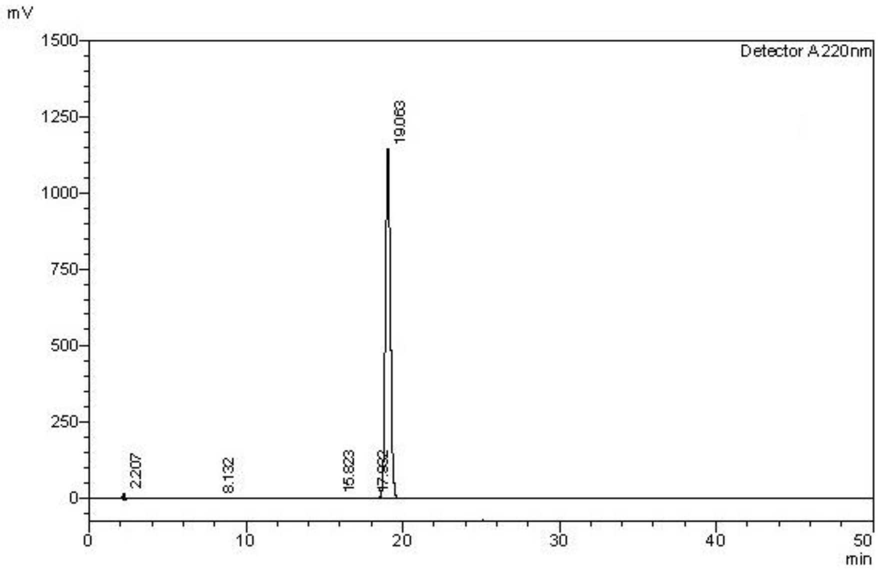

<Peak Table>

Detector A 220nm

Peak#	Ret. Time	Area	Area%	Height	tical Plates/mete
1	2.207	126461	0.510	19650	19741
2	8.132	3415	0.014	351	132585
3	15.823	15027	0.061	1006	131581
4	17.932	3526	0.014	205	95991
5	19.063	24643514	99.401	1144638	119765
Total		24791942	100.000	1165852	

G:\220nm\NST15.lcd

HPLC purity (NST6A-D)
